# A Multinational Longitudinal Study Incorporating Intensive Methods to Examine Caregiver Experiences in the Context of Chronic Health Conditions: Protocol of the ENTWINE-iCohort

**DOI:** 10.3390/ijerph19020821

**Published:** 2022-01-12

**Authors:** Val Morrison, Mikołaj Zarzycki, Noa Vilchinsky, Robbert Sanderman, Giovanni Lamura, Oliver Fisher, Giulia Ferraris, Saif Elayan, Erik Buskens, Eva Bei, Anne Looijmans, Viola Angelini, Mariët Hagedoorn

**Affiliations:** 1 School of Human and Behavioural Sciences, Bangor University, Bangor LL57 2AS, UK; zarzycki@bangor.ac.uk; 2 Department of Psychology, Faculty of Social Sciences, Bar-Ilan Univeristy, Ramat Gan 5290002, Israel; noa.vilchinsky@biu.ac.il (N.V.); eva.bei@biu.ac.il (E.B.); 3 Department of Health Psychology, University Medical Center Groningen, University of Groningen, 9713 GZ Groningen, The Netherlands; r.sanderman@umcg.nl (R.S.); g.m.a.ferraris@umcg.nl (G.F.); a.looijmans@umcg.nl (A.L.); mariet.hagedoorn@umcg.nl (M.H.); 4 Centre for Socio-Economic Research on Ageing, IRCCS INRCA-National Institute of Health and Science on Ageing, 60124 Ancona, Italy; g.lamura@incra.it (G.L.); o.fisher@inrca.it (O.F.); 5 Department of Economics and Social Sciences, Università Politecnica delle Marche, 60121 Ancona, Italy; 6 Department of Economics, Econometrics and Finance, Faculty of Economics and Business, University of Groningen, Nettelbosje 2, 9747 AE Groningen, The Netherlands; s.y.i.elayan@rug.nl (S.E.); v.angelini@rug.nl (V.A.); 7 Faculty of Medical Sciences, Antonius Deusinglaan 1, 9713 AV Groningen, The Netherlands; e.buskens@umcg.nl

**Keywords:** informal caregiving, multinational, caregiver motivations, wellbeing

## Abstract

Informal caregivers are those who provide unpaid care to a relative or friend with a chronic illness, disability or other long-lasting health or care need. Providing informal care in the context of chronic health conditions presents a significant global challenge. Examination of the determinants of informal caregivers’ behaviour, especially in terms of motivations and willingness to provide/receive care, is crucial to understanding the nature of caregiver and care recipient experiences. A large group of international researchers have co-operated to execute the ENTWINE iCohort-a multinational, transdisciplinary, longitudinal study incorporating intensive methods to examine caregiver experiences in the context of chronic health conditions. The aim of ENTWINE-iCohort is to investigate the broad spectrum of factors, i.e., cultural, personal, geographical, relational, psychological, and economic that may affect motivations, willingness to provide or receive care, among diverse groups of informal caregivers and their care recipients, in different countries that have different care systems. Study questionnaires will be disseminated on-line in nine countries: Germany, Greece, Ireland, Italy, Israel, the Netherlands, Poland, Sweden, and the UK. Cross-sectional and longitudinal multivariate analysis, including intensive longitudinal and dyadic data analysis will be applied to examine the relative contribution of the above factors to caregiver or care recipient wellbeing.

## 1. Introduction

Due to medical advancements and the subsequent rise in longevity worldwide, societies everywhere are facing a growing ageing population with an increase in those living with chronic, often disabling health conditions. However, in spite of this, many countries, such as the United Kingdom, The Netherlands and Italy, have seen a decline in spending on social care over the past decade [1,2]. Although investment has taken place more recently adult social care is considered to be in crisis and a significant funding gap exists [1,2]. Whilst investment in social care services fails to meet demand, families are required to manage care more informally, with the burden of care typically falling to female family members [3,4]. This continuing gender imbalance is in spite of an increased percentage of women who are also in paid employment [5,6]. This means that where the needs of older/ill individuals for long-term care is rising, the availability of informal caregivers is in decline. Known as the “Care Gap”, this will create huge problems for the sustainability of global health care systems that rely heavily on the provision of informal care. Thus, a multinational analysis of informal caregivers’ willingness to care and putative determinants of it as well as of informal caregivers’ wellbeing and other outcomes is essential for informing care solutions and public policy. 

A large group of international researchers have co-operated to execute the ENTWINE iCohort—a multinational, transdisciplinary longitudinal study incorporating intensive methods to examine caregiver experiences in the context of chronic health conditions. This paper presents the study protocol.

An informal caregiver for ENTWINE purposes is defined as a family member or friend who adopts a caring role for a person with a chronic health condition, disability, or other care need where needs require the caregiver to perform tasks beyond those typical of their usual role/relationship.

Research has commonly identified love and affection as the primary motivation for adopting a typically unpaid caregiver role [7,8] along with motives of duty and reciprocity [9]. However the complexity of dyadic relationships and attachments [10,11,12,13,14], the existence of varying cultural norms, values and obligations [15,16], competing social and economic demands on potential caregivers [17] and the fact that modern families tend not to be as geographically co-located as in previous generations [18,19,20,21,22] are less typically examined in relation to their influence on caregiver motivations. We face major challenges if we are to meet the demand for care (including informal care) across Europe on a societal (and often familial) assumption of family member willingness to adopt a caregiving role in the home or community. There is a real risk that the numbers experiencing negative physical and mental wellbeing as a consequence of caregiving [23,24,25,26,27] will increase and/or that the willingness to care will subside as people are expected to work for longer [28,29,30].

If informal care is to be maintained and supported, we need to better understand the range of factors—personal, interpersonal, social, economic, and geographic—that may influence caregiver willingness and motivation to adopt and maintain this demanding role or which may mitigate against negative care outcomes. The evidence gap that this study seeks to address will be informed by data from multiple countries facing similar ageing demographics but with different cultural and social norms around illness and care, and different care systems. Such understanding is important to the creation of culturally relevant policy and practice.

### 1.1. Primary Objective

To examine the current experience of caregiving, motivations to care and influences thereon among a diverse group of informal caregivers recruited by means of an intensive longitudinal cohort study (ENTWINE-iCohort) conducted in the five countries represented in the ENTWINE Consortium network (UK, Ireland, The Netherlands, Italy and Israel) plus four other EU countries (Germany, Greece, Poland, Sweden).

### 1.2. Secondary Objectives

To conduct a full examination of the:

Determinants of, and cultural differences in, motivations and willingness to provide care with specific attention paid to the contribution of personal values and meanings attached to illness and caregiving and their impact on caregiver outcomes,

Personal and geographical barriers and facilitators of caregiving with specific attention paid to the contribution of caregivers’ personality traits and geographic distance from the care recipient upon willingness to care and caregiver outcomes,

Diversity in experiences of caregiver—care recipient dyads, with specific attention paid to the characteristics of the dyads, interpersonal processes, willingness to provide and receive care and individual and relational outcomes.

Patterns of and differences across countries in informal care costs, as well as the preferences for, and use of, formal versus informal caregiving, and the personal and contextual influences thereon.

Characteristic of access to, use of and challenges of household-based migrant care work and how this supports informal caregiving

Each of these objectives is addressed in detail by one early stage researcher (five in total), each under supervision of two senior academics, and with oversight held by a Work Package Lead and ENTWINE management team.

## 2. Materials and Methods

Full study design:

The ENTWINE iCohort study shall combine:

(i) longitudinal data collection (Baseline + 6 months follow-up) using an electronic survey tool with measures and items addressing the project objectives, with a sample of informal caregivers;
(ii) longitudinal data collection (Baseline + 6 months) using an electronic survey tool containing a subset of measures and items addressing the project objectives, with a sample of care recipients;
(iii) a weekly assessment component to examine change over time using a subset of measures employed with caregivers and care recipients.

Setting:

Countries directly involved in the WorkPackage, ENTWINE iCohort included the UK (Morrison, Zarzycki); The Netherlands (Hagedoorn; Buskens, Ferraris, Elayan); Israel (Vilchinsky, Bei); and Italy (Lamura, Fisher) and the early stage researchers appointed also brought representation from Greece and Poland. Three further countries joined as collaborators based on targeting geographical diversity across Europe (Ireland, Germany, Sweden), and thus the setting was inclusive of northern, southern, eastern and western Europe, and affiliated country Israel.

The QUESTBACK online system was selected for collecting and storing data for the ENTWINE iCohort. Questback EFS (Enterprise Feedback Suite) is recognized by the Coordinating Centre UMCG as a secure data sharing tool. Data in EFS is only accessible by the members of the research team and consists of the e-mail address participants provide, their code and the data collected in the questionnaires. As such, this platform was considered suitable for our multinational study.

The Sample Selection Inclusion/Exclusion

There will be two samples: Caregivers and a care recipient sub-sample to enable dyadic analyses for those matched to a caregiver.

Inclusion criteria:

To be eligible, caregivers must:

Be aged over 18 years old;

Be providing care for a family member or friend with a chronic health condition, disability or any other care need;

Provide self-declared cognitive capacity to complete questionnaires (brief screener included to check for cognitive impairment);

Have access to the Internet.

The secondary sample is that of care recipients, identified either by a participating caregiver as receiving their care, or through direct recruitment advertising. To be eligible, care recipients must

Be aged over 18 years old;

Have a caregiver declaration regarding care recipient cognitive and physical capacity to complete questionnaires online;

Have access to the Internet.

## 3. Recruitment Methods

Caregivers and care recipients are invited to participate jointly in the intensive longitudinal cohort study, but caregivers are encouraged to participate even where the care recipient is unable/unwilling to participate.

Recruitment will be either by (1) broad-based online recruitment via academic, social and news media and care or patient relevant organisations or (2) a more targeted, direct approach via user groups, carer organisations and primary and secondary care settings using advertising (poster displays, flier distribution) and direct face-to-face contact (due to COVID-19 restrictions direct face-to-face recruitment was not initiated). For recruitment method 1 a multi-language advertisement/flier was produced for all included study sites inviting adults who meet the aforementioned eligibility (inclusion) criteria to, for example,

“Take part in an international study of caregiving—find out more at this URL for ENTWINE iCohort survey link” (see Supplementary File S1).

These fliers shall be distributed in each country via national and international charity organisations specific to our target conditions, e.g., Alzheimer’s Society, Stroke Association, Eurocarers (and national equivalents) and, subject to local approvals, also displayed in public venues such as community pharmacists, health, leisure, or educational centres.

The flier/advertisement will direct potential caregiver participants to the online platform for the ENTWINE-iCohort where they can register and access an eligibility survey (Supplementary File S11), a full Participant Information Sheet (PIS, Supplementary File S2) and electronic consent (Consent Form, Supplementary File S3) shall be required before the caregiver survey (baseline or follow-up, or the intensive longitudinal component, Supplementary Files S4, S6 and S8) can be accessed.

The caregiver (CG) shall be invited to consider whether they wish to nominate their care recipient (CR) to take part in the parallel Care Recipient Survey (baseline or follow-up, or the intensive longitudinal component, Supplementary Files S5, S7 and S9) and if so they shall direct them to the relevant registration site where they can access the CR survey tool for independent completion. CRs who self-identify will also be directed to the relevant registration site to access the CR survey tool. Both CG and CR will also be asked to indicate willingness to be contacted for a future related research study (Supplementary File S10).

## 4. Questionnaire Randomisation

**Figure 1:**
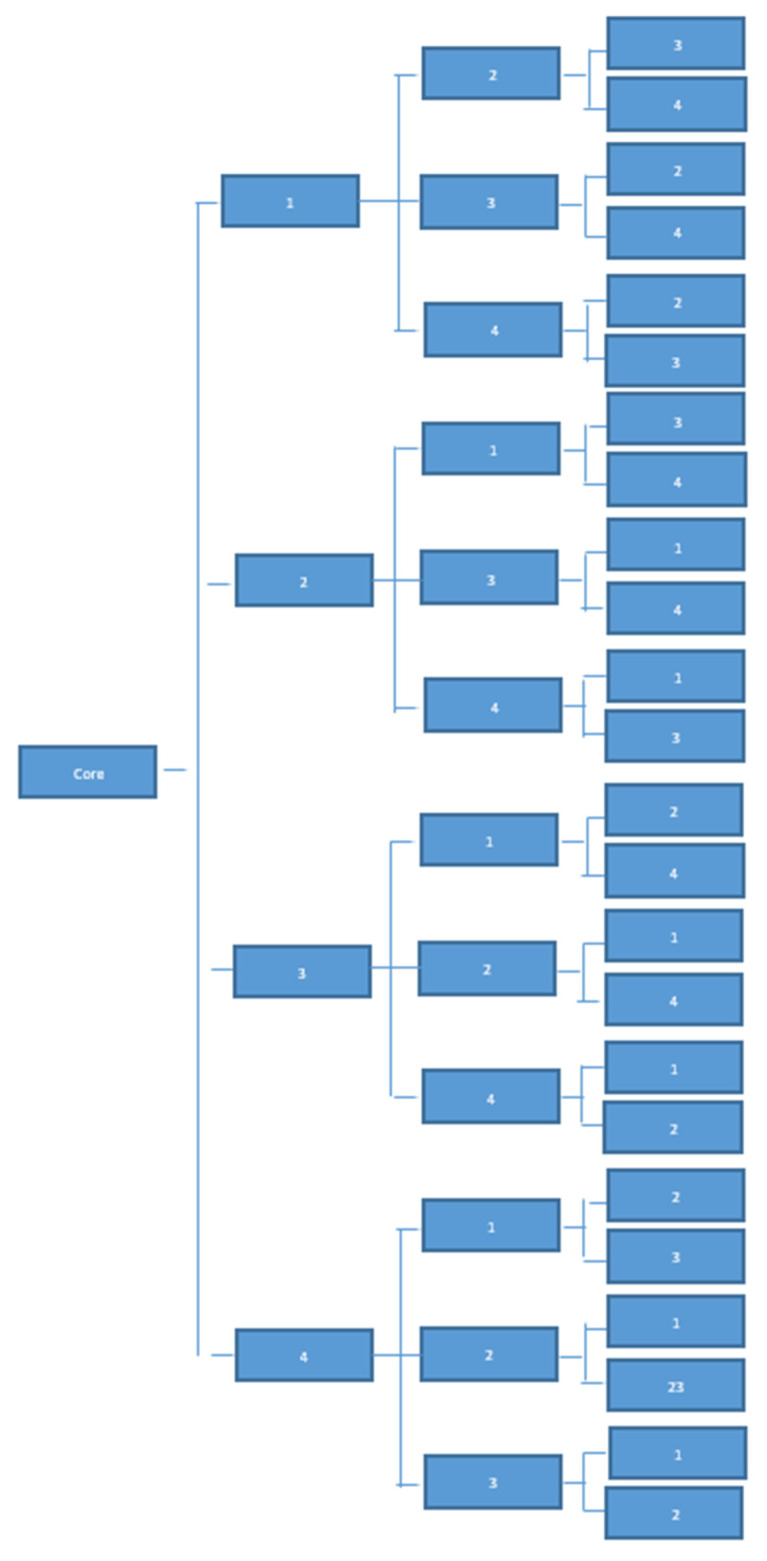
Questback randomization process. Note: 1 = cultural and psychosocial aspects; 2 = personality and geographical barriers; 3 = interpersonal processes; 4 = employment, costs and migrant care work.

Caregivers: the questionnaire for the CG is divided into four modules (Note that the economic, employment and migrant care work sub-components were combined into one module although this addresses the work of 2 ESRs), that is a core module and one module for variables that are specific for each sub-study (1 = cultural aspects; 2 = personality and geographical barriers; 3 = interpersonal processes; 4 = employment and costs including migrant care work). The core module includes personal and demographic information, care context and care task information, willingness to care and wellbeing outcomes (affect, gains, burden). In addition to the core module, participants will randomly receive two other modules (see Figure 1) as programmed within Questback.

This process will reduce the burden for participants and ensures that drop-out and missing values do not centre on specific modules (i.e., if a module is always offered last, this could increase the chance of missing data for this specific module).

Care recipients. The questionnaire for the CR is much shorter and the same for all CRs i.e., there are no modules.

## 5. Sample Size Calculation

Whilst each specific ESR project has its own objectives (as described above), three are addressing those in relation to the primary outcomes of willingness and motivations to care. Given the number of variables to be assessed (see Measures, approximately 30 variables will be used in total) it is our intention to recruit CGs to the case: variable ratio of 10:1, i.e., 300 CGs per country. For CRs, who are assessed on fewer variables, we expect at least 150 participants per country—anticipating that at least 50% of our sample of 300 CGs per country will identify an eligible care recipient (based on our previous research). Analyses will be appropriate to the attained sample size on study completion in order to achieve satisfactory statistical power.

The a priori sample size calculation was informed by previous research. Achieving 90% power to detect a medium sized association/ explained variance R^2^ = 0.25, with entry of up to 10 independent variables (randomly selected variables dependent on prior analyses) and willingness to care (measured as a continuous variable) in a multivariate regression analysis with an alpha of 5% (G*Power 3.1, [31]).

## 6. Measures

Table 1 presents all measures used within the ENTWINE iCohort survey, with a brief description of the concept addressed, timings of use, and whether they reflect a key outcome measure (DV) or an IV/potential mediator or moderator variable. Wherever possible, validated instruments were employed, and short-form assessments were preferable where available in order to reduce participant burden.

Measures that are not validated and available in the required language will be translated into the appropriate languages using accredited translators who will be native speakers of the target languages and fluent in English. Translations will be checked for compatibility with the original version in a process of back translation, also performed by persons who will be native in the foreign language and fluent in the English language, to ensure that none of the original meaning is lost. For each language, a potential research consultant/reviewer will be identified to ensure that any discrepancies between the forward and back translations can be resolved appropriately by discussion with the translators. All translations will be coordinated by one project partner to ensure consistency. Piloting in each country shall enable identification of any semantic inconsistencies.

## 7. Analysis Plan

Five separate studies are addressed, and each will use selected variables from the Core survey, plus module-specific items. For each study, baseline data will first be analysed for the full sample, with a smaller sample entering longitudinal analysis dependent on completing both timepoints of the survey. The analysis plan will evolve depending on our a priori set of research questions, as well as the sample characteristics and final sample size.

*Missing data handling.* Prior to analysis the data set will be cleansed and checked for missing data. Missing data are a critical challenge when dealing with online surveys thus the ENTWINE iCohort survey will employ multiple checkpoints to address this. In the cases where missing data exist, a descriptive analysis of the missing data, as well as a number of hypothesis tests, will be carried out to determine the mechanism of missingness. These tests include Little’s test and Fairclough’s logistic regression method [59,60]. If data are missing completely at random (MCAR), missing values will be subjected to list-wise deletion and complete case analysis will be performed. If data is missing at random (MAR), we will adopt a hot deck imputation for variables with a negligible proportion of missing values (i.e., less than 5%) and multiple imputations by chained equations (MICE) for variables with a larger proportion of missing values [61,62,63,64].

For all analyses, assumptions regarding multicollinearity, singularity, normality, linearity, and homoscedasticity will be tested.

### 7.1. Modules 1-2-3 Plan Analyses

Descriptive analyses will be performed to establish frequencies, means and standard deviations across key categorical and continuous variables of interest. Bivariate and multivariate analyses, including analysis of variance, *t*-tests and correlations will examine the relationship between caregiver and care recipient characteristics (e.g., age, gender, ethnicity, illness type, type of caregiving relationship, etc.), willingness and motivations to provide care, and key caregiver and care recipient outcomes (e.g., caregiver burden, depression, relationship satisfaction etc.).

Relevant, theoretically informed tests of mediation and moderation will be applied prior to conducting hierarchical multivariate regression analyses to assess the extent of outcome variance explained by the IV set, for example moderating or mediating the effects of: (1) relationship quality, gender, or perceived choice in assuming the caregiver role; (2) willingness to care and relationship quality of the caregiving dyad; (3) interpersonal processes and type of relationship between caregiver and care recipient.

Changes over time in IVs or caregiving outcomes will be analysed in the longitudinal data set using repeated measures tests (*t*-tests, ANOVA, MANOVA).

### 7.2. Module 4 Plan Analysis

Descriptive analyses will be conducted with sociodemographic and caregiving situation data to summarise sample characteristics, informal care costs and the use of paid home care services.

Informal care costs including employment-related costs, caregiver-time costs, and out-of-pocket expenses will be estimated. Unlike out-of-pocket costs, time- and employment-related costs are not measured in monetary terms and, therefore, should be valued. Time costs will be valued by two methods: the opportunity costs and the proxy good (following the recommendation in van den Berg et al. [65]). Additionally, employment-related costs will be valued using the human capital approach. Additional informal caregivers, the underlying level of dependency (i.e., need) of, and distance to, the care recipient may mediate the time and burden perceived. During analyses these interactions will be explored. Furthermore, multivariate regression analysis will be performed to find the determinants of informal care costs, e.g., those mentioned above, the demographic and socioeconomic characteristics of both the caregiver and care recipient, as well as the use of national or local formal care and support services.

The impact of the hiring of paid care workers (alongside other factors) on the amount of informal care will be examined using bivariate and linear regression. Furthermore, binary logistic regression will be used to calculate the predictors (including the hiring of paid care workers) of not having a low level of subjective wellbeing (score of 50 or over on the WHO-5 index). Finally, hours of personal care, household activities, and practical support by informal caregivers and paid care workers will be cross tabulated with each other to determine if care tasks are provided by both informal caregivers and paid care workers, only provided by paid care workers or informal caregivers, or provided by neither.

## 8. Weekly Assessment Component

In the weekly assessment component, multilevel modelling will be used to account for the nested data structure (i.e., weekly diary assessments nested within caregivers [66]) in order to assess within-person main, interaction and moderation effects. Intensive longitudinal designs (e.g., weekly assessments) can better provide critical information on fluctuations in willingness to care, and identify how they are associated with changes in care tasks, caregiver burden or mood and in interpersonal processes between caregivers and care recipients (e.g., dyadic coping behaviours). These data will examine time-lagged associations between key factors and caregiver outcomes in order to gain better insight into the sequence of events and address the predictive utility of our measures in relation to key outcomes. Our analyses will tease apart the specific challenges faced by informal caregivers at different stages of the disease of the care recipient, i.e., during different periods in their caregiving role, and thus help to target support (e.g., eHealth) at the appropriate time.

## 9. Dissemination

A dissemination strategy has been prepared by the ENTWINE WorkPackage group and our Dissemination and Website Taskforce. This will include dissemination to both academic and non-academic stakeholders in health, public health and social care sectors by means of conference presentations, blogs and multi-media news items on our study website, and preparation of a newsletter providing an executive summary of study findings and their implications.

**Study status**-Baseline recruitment to 31 August 2021; recruitment figures 2736 recruited, 1834 completers (1120 full completion) with follow-up ongoing.

Ireland 14/08/2020–31/05/2021
UK 14/08/2020–31/05/2021
Poland 14/08/2020–31/05/2021
Italy 25/08/2020–31/05/2021
Netherlands 16/10/2020–31/05/2021
Sweden 23/10/2020–31/05/2021
Greece 31/10/2020–31/05/2021
Israel 16/02/2021–31/08/2021
Germany 17/02/2021–31/08/2021

Participants were invited to take part in the follow up assessment 181 days after the initial baseline invitation. Data collection completed for all countries on 15 December 21; 6 month follow-ups were not offered to participants who completed baseline data after 31 May 2021 due to project funding deadline restrictions.

## Figures and Tables

**Table 1:** Summary of measures.

Section/Measure	Description and Max*N of Items	Scoring	IV, DV, Mod, Med	CG Baseline	CG Follow Up	CR BASELINE	CR Follow Up
**Core**							
Caregiver Self-report Personal and Demographic Questionnaire	Seventy four (74) items addressing age, gender, home location, partnership status, relationship type, religion, ethnicity, household composition, highest level of attained education, employment status, income, other dependents, own health condition, health condition of care recipient, length of illness, distance from caregiver to care recipient, treatment or medication,	Categorical responses	IV, Mod, Med	✔	✔	✘	✘
Care Recipient Self-report Personal and Demographic Questionnaire	Fifty eight (58) items addressing age, gender, home location, partnership status, relationship type, religion, ethnicity, highest level of attained education, employment status, income, other dependents, own health condition, health condition of care recipient, length of illness, distance from caregiver to care recipient	Categorical responses	IV, Mo, Me	✘	✘	✔	✔
Inclusion of Other Self Scale (IOS) [32]	One item assesses the extent to which participants include another person in their sense of self	7-point Likert scale in which participants choose a pair of circles from seven with different degrees of overlap (ranging from 1 = no overlap; to 7 = most overlap) Higher scores indicate greater inclusion of other in self	IV, Mod, Med	✔	✔	✔	✔
Katz ADL [33]	Six items assess functional status via measurement of the person’s ability to perform activities of daily living independently	Items address independence yes (1), no (0) in performing bathing, dressing, toileting, transferring, continence, and feeding. A score of 6 indicates full function, 4 indicates moderate impairment, and 2 or less indicates severe functional impairment.	IV	✔	✔	✔	✔
COVID-19 related questions	Ten (10) items address the impact of COVID-19 on employment, access to support services, willingness to care, provision of practical, emotional, personal care	Numerical and categorical	Med, Mod	✔	✔	✘	✘
Use of paid home care services and their characteristics	Thirty five (35) items in the caregiver baseline and follow up and 37 items in the care recipient baseline and follow up assess the self-reported use of paid home care services by the care recipient. Demographics of paid care workers i.e., gender, age, nationality, migration background, live-in or live-out	Numerical and categorical	IV, DV	✔	✔	✔	✔
Financial benefits	Six items regarding: cash benefits, financial compensation during care leave, tax benefits e.g., exemptions, deductions, credits, coverage of social or pension contributions, caregiver credits, and health insurance	Numerical and dichotomous questions	IV, Mod/Med	✔	✔	✘	✘
Motivations in Elder Care Scale (MECS) [34]	Two item sub-scales: Extrinsic Motivations to Care (EXMECS) and Intrinsic Motivations to Care (INMECS)	5-point scale ranging from 1 (Strongly disagree) to 5 (Strongly agree). A higher score indicates greater motivations to provide care.	IV, DV, Med, Mod	✔	✔	✘	✘
Partner-Specific Communal Motivation Scale (CMS) [35]	Ten (10) items address communal motivation to care	9-point scale ranging from 1 (Extremely disagree) to 9 (Extremely agree). A high score reflects greater CM. (items 2, 5 and 10 are reverse scored before summing)	IV	✔	✔	✔ adapted	✔ adapted
Willingness to Care Scale [36]	Thirty (30) items assess willingness to provide emotional, instrumental, and nursing care related tasks	5-point Likert scale (1 = completely unwilling to complete the task, 5 = completely willing)	IV, DV, Med, Mod	✔	✔	✘	✘
Willingness to Receive Care, adapted from Abell [36]	Three items assess willingness to receive emotional, instrumental, and nursing care-related tasks	5-point Likert scale (1 = completely unwilling to complete the task, 5 = completely willing)	IV, DV, Med, Mod	✘	✘	✔	✔
The World Health Organisation- Five Well-Being Index (WHO-5) [37]	Five items assess caregiver/care recipient well-being	6-point scale ranging from 0 (at no time) to 5 (all of the time). High scores indicate greater wellbeing	DV	✔	✔	✔	✔
The GAINS Scale [38]	Ten (10) items assess perceived gains associated with caregiving	Items measured on a 4-point Likert scale from 0 = Not at all to 3= A lot, with a possible maximum score of 30. Higher scores indicate greater gains.	DV	✔	✔	✘	✘
Short Form Zarit Burden Interview (ZBI-12) [39]	Twelve (12) items assess caregiver burden	5-point Likert scale ranging from 0 = Never to 4 = Always Total scores range from 0 to 48 with higher scores (>20) indicating high levels of burden	DV	✔	✔	✘	✘
EQ-5D-5L and EQ VAS [40]	Five dimensions of health state are assessed using 5 items: mobility, self-care, usual activities, pain/discomfort and anxiety/depression. The EQ VAS is a single item that records the patient’s self-rated health on a vertical “ladder” visual analogue scale.	Each dimension has 5 levels: no problems, slight problems, moderate problems, severe problems and extreme problems. The VAS endpoints are labelled ‘The best imaginable health state (100)’ and ‘The worst imaginable health state (0)	IV, DV	✔	✔	✔	✔
Centre for Epidemiological Studies Depression Scale (CESD-10) [41,42]	This 10-item screening tool assesses depressive symptoms in the past week	4-point Likert scale ranging from 0 = Rarely or none of the time to 3 = All the time. (items 5 and 8 are reverse scored before summing)	IV, DV	✔	✔	✔	✔
Dyadic Relationship Scale (DRS) [43]	The 11-item scale for the caregiver baseline and follow up and the 10 item scale for the care recipient baseline and follow up assess positive dyadic interactions and negative dyadic strain experienced by caregivers and care recipients.	5-point Likert scale ranging from 0 = strongly agree to 4 = strongly disagree. Reversed items: 3,4,5,8,11. Higher scores indicate higher levels of strain and positive interaction.	DV	✔	✔	✔	✔
Relationship satisfaction (RAS) [44]	A single item measure of relationship satisfaction	5-point Likert scale ranging from 1 = not satisfied to 5 = very satisfied	DV	✔	✔	✔	✔
Caregiver Indirect and Informal Care Cost Assessment Questionnaire [45]	Twelve (12) items enable calculation of estimated indirect (productivity) and informal care costs as mutually exclusive subsets of total costs		DV, IV	✔	✔	✘	✘
**Module 1**							
Revised Familism Scale (RFS) [46]	Twenty one (21) items across three sub-scales: Familial interconnectedness; Familial obligations; Extended family support.	5-point Likert scale, with 0 indicating ‘very much in disagreement’ and 4 ‘very much in agreement.’	IV	✔	✔	✔	✔
Brief Illness Perception Questionnaire (B-IPQ) [47]	Nine items, with a single item each assessing illness consequences, timeline, personal control, treatment control, identity, coherence, emotional representation, and illness concern	Each item assessed on a scale from 1 to 10 (modified response range). A summed score represents the degree to which the illness is perceived as threatening.	IV	✔	✔	✔	✔
Meaning in Life Questionnaire (MLQ) [48]	Five items each assess two dimensions of meaning in life: (1) Presence of Meaning (2) Search for Meaning.	7-point Likert scale from 1 (‘absolutely untrue’) to 7 (‘absolutely true’).	IV	✔	✔	✔	✔
Portrait Values Questionnaire (PVQ-21) [49]	Nine items assess altogether two subscales of personal values: Self-transcendence and Self-enhancement.	6-point Likert response scale from “very much like me” (1), to “not like me at all. (6).” The subscale score is obtained by calculating the mean of the relevant item scores.	IV	✔	✔	✔	✔
Perceived choice in assuming the caregiving role	Single item: Do you feel you had a choice in taking on this responsibility of caring for your loved one?	Yes/No	IV	✔	✔	✔	✔
The importance of religion	Single item: What is the importance of religion in your life?	4-point response scale from 1 (“not important at all”) to 4 (“very important”)	IV	✔	✔	✔	✔
**Module 2**							
The geographies of care	Thirty (30) items for the CG survey and 8 for the CR identify two aspects of caregiving geographies; a) setting, access, characteristics; and b) perceived geographical barriers and facilitators to informal care provision.	Mixed format, Likert scale responses and dichotomous questions (Yes/No)	IV	✔	✔	✔	✔
Big-Five Inventory Extra Short Form (BFI-2-XS) [50]	15 items measure the domains of Extraversion, Agreeableness, Conscientiousness, Neuroticism, Openness to Experience	5-point Likert scale ranging from 1 = Disagree strongly to 5= Agree strongly.	IV	✔	✔	✔	✔
The Relationship Structures Questionnaire of the Experiences in Close Relationships-Revised (ECR-RS) [51]	Nine items assess (1) attachment-related anxiety and (2) attachment-related avoidance	4-point Likert scale ranging from 1 = Strongly Disagree to 4 = Strongly Agree. Scores are computed for each of the two subscales by averaging item responses.	IV	✔	✔	✔	✔
Toronto Empathy Questionnaire (TEQ) [52]	Sixteen (16) items assess empathy as a primarily emotional process.	5-point Likert scale ranging from 0 = Never to 4 = Always. Scores below 45 are indicative of below average empathy levels.	IV	✔	✔	✔	✔
The Pearlin Mastery Scale [53]	This seven-item scale assesses the extent to which an individual regards their life chances as being under their personal control	4-point Likert scale ranging from 1 = Strongly Disagree to 4 = Strongly Agree	IV	✔	✔	✔	✔
**Module 3**							
Perception of Collaboration Questionnaire (PCQ) [54]	This nine-item scale assesses three dimensions of collaboration between caregiver and care recipient: (1) Cognitive Compensation (2) Interpersonal Enjoyment and (3) Frequency.	5-point Likert scale ranging from 1 = strongly disagree to 5 = strongly agree with higher scores indicating stronger agreement. Items 5 and 9 are reverse scored.	IV, Me	✔	✔	✔	✔
Dyadic Coping Inventory (DCI)-communication subscale [55]	The eight-item DCI measures perceived communication and dyadic coping within a close relationship when one or both dyad members are stressed.	5-point scale from 1 = very rarely to 5 = very often. Subscale scores include: (a) Stress communicated by oneself (SCO: items 1, 2, 3, and 4); (b) Stress communication of the partner (SCP: items 5, 6, 7 and 8).	IV, Me	✔	✔	✔	✔
Mutuality Scale (MS) [56]	Fifteen (15) items measure mutuality from either the caregiver or the care recipient perspective, across four dimensions: love and affection, shared pleasurable activities, shared values, and reciprocity.	5-point Likert scale ranging from 1 = not at all to 4 = a great deal. A total scale score is computed by averaging all item scores.	IV, Me	✔	✔	✔	✔
The perceived partner responsiveness scale (PPRS) [57]	The 12-item PPRS measures the degree to which people feel that their significant others are responsive to them.	9-point Likert scale ranging from 1 = not at all true to 9 = completely true.	IV, Me	✔	✔	✔	✔
Social Support List (SSL) [58]	Six items measure perceived supportive behaviours and seven items measure perceived unsupportive behaviour from the caregiver and the care recipient perspective.	4-point Likert scale ranging from 1 = rarely or never to 4 = very often. The scores are summed into two indexes, with a higher score indicating a higher frequency of supportive and unsupportive behaviour.	IV, Me	✔	✔	✔	✔
**Module 4**							
The influence of informal care on employment and the associated costs.	Seven self-reported items address the influences of informal care on the employment situation of the caregiver, and the associated costs of this.	Categorical and numerical answers	IV, DV	✔	✔	✘	✘
Types of home care services provided by paid care workers	Eighteen (18) items assess which tasks and how many hours of care tasks (total and per type of care task) are provided by paid home care workers	Categorical and numerical answers.	IV, DV	✔	✔	✘	✘
Rationale for hiring of paid care workers	Twenty two (22) items assess the rationale for hiring paid home care workers and the decision to hire or not hire migrant care workers	Categorical answers, with each item treated separately	DV	✔	✔	✔	✔
Out-of-pocket expenses incurred by caregivers as a result of their caregiving role.	Twenty five (25) items measure out-of-pocket costs for caregivers (both in terms of overall total and per type of cost). These costs include expenditure on, e.g., caregiving support services, medical care, food, travel, home care services, aids, appliances and home modifications due to care recipient’s condition.	Categorical and numerical answers.	IV, DV	✔	✔	✘	✘
Care benefits received by the care recipient	Four self-reported items measure care benefits received (both in terms of overall total and per type of care benefit), e.g., tax benefits, cash benefits, care vouchers.	Categorical and numerical answers.	IV, DV	✘	✘	✔	✔
Self-reported questionnaire on the use of, and the out-of-pocket expenses for, care services, as well as assistive devices and aids used by the care recipient.	Twenty two (22) items detail which types of support services the care recipient receives in relation to their care and the out of pocket expenses spent in relation to their care.	Categorical and numerical answers	IV, DV	✘	✘	✔	✔

*Legend:* CG = caregiver; CR = care recipient; IV = independent variable; DV = dependent variable; Mo = moderator; Me = mediator.

## Data Availability

This is a protocol paper however all anonymized data will be openly available upon request after September 2023, or on completion of ENTWINE teams key outputs if this is earlier. OpenAIRE and Zenodo will be used as data repositories.

## Supplementary Materials

The following are available online at https://www.mdpi.com/article/10.3390/ijerph19020821/s1, Supplementary File S1: ENTWINE-iCohort exemplary fliers (1–3); Supplementary File S2: Participant Information Sheet for ENTWINE-iCohort Prospective Study in Informal Caregiving; Supplementary File S3: Participant Consent Forms to participate in the survey; Supplementary File S4: ENTWINE-iCOHORT Caregiver Baseline Survey; Supplementary File S5: ENTWINE-iCOHORT Care Recipient Baseline Survey; Supplementary File S6: ENTWINE-iCOHORT Caregiver Follow up Survey; Supplementary File S7: ENTWINE-iCOHORT Care Recipient Follow up Survey; Supplementary File S8: ENTWINE-iCOHORT Diary Study Weekly Measures-Caregiver version; Supplementary File S9: ENTWINE-iCOHORT Diary Study Weekly Measures-Care Recipient; Supplementary File S10: Consent to be contacted for future studies; Supplementary File S11: Participant Consent Form to be contacted for future studies; Eligibility Survey.
