# Supplementary material for: A Multinational Longitudinal Study Incorporating Intensive Methods to Examine Caregiver Experiences in the Context of Chronic Health Conditions: Protocol of the ENTWINE-iCohort
Source: Int J Environ Res Public Health. 2022 Jan 12;19(2):821. doi: 10.3390/ijerph19020821 (PMC8775526; doi:10.3390/ijerph19020821)
Supplement: Supplementary file 1 [file ijerph-19-00821-s001.zip › Supplementary File S10. Consent to be contacted for future studies.pdf]

Consent to be contacted for future studies

Title of Project: ENTWINE Prospective Cohort Study in Informal Care (ENTWINE-iCohort)

Names of Researchers: Mikołaj Zarzycki, Eva Bei, Giulia Ferraris, Saif Elayan and Oliver Fisher

By signing this consent form, you agree to the following statements:

1. I have been told about the possibility of being contacted for future studies. I understand that my participation in these future studies is voluntary and that my refusal to participate will not affect my participation in the main study.

2. I understand that my email address will be stored securely by the study staff for this purpose and only for the 3-year duration of the ENTWINE Project. No information will be collected about me without my consent.

3. I may only be contacted for studies that relate to this study's subject area over the next three years by email. I am not obligated to participate in any research studies that I am contacted about.

4. I am not consenting to have additional measurements taken but to be contacted later on. If I am contacted for a future research study, I will still have the opportunity to decline at that time, and if I decide I am interested, I will be asked to sign a separate consent form for each individual study.

☑ I have read the above information, and I agreed to be contacted for future research as described above.

This form will be produced in duplicate. One digital copy should be retained by the participant and the other will be sent electronically to the ENTWINE iCohort management team.
