# Supplementary material for: A Multinational Longitudinal Study Incorporating Intensive Methods to Examine Caregiver Experiences in the Context of Chronic Health Conditions: Protocol of the ENTWINE-iCohort
Source: Int J Environ Res Public Health. 2022 Jan 12;19(2):821. doi: 10.3390/ijerph19020821 (PMC8775526; doi:10.3390/ijerph19020821)
Supplement: Supplementary file 1 [file ijerph-19-00821-s001.zip › Supplementary File S11. Participant Consent Form to be contacted for future studies; Eligibility Survey.pdf]

Participant Consent Form to be contacted for future studies; Eligibility Survey

1. Please select from the list below the language you would like to take this survey in.

- ❑ English
- ❑ Dutch
- ❑ Greek
- ❑ Italian
- ❑ Polish
- ❑ Swedish
- ❑ German
- ❑ Hebrew

2. Please select from the list below your current country of residence.

- ❑ UK
- ❑ The Netherlands
- ❑ Greece
- ❑ Italy
- ❑ Ireland
- ❑ Poland
- ❑ Sweden
- ❑ Germany
- ❑ Israel

3. How did you hear about this study?

- ❑ Flyer/leaflet or poster display (please provide location)
- ❑ A Family member or friend
- ❑ Health care provider
- ❑ Facebook post/group
- ❑ Twitter post
- ❑ Other social media

- ❑ Email
- ❑ Radio
- ❑ Newspaper
- ❑ Word of mouth
- ❑ Caregiver or patient support group
- ❑ ENTWINE study website
- ❑ Other

Before we can tell you more about the study, we have to find out whether you are eligible to participate. In order to find out, please answer the following questions. By filling in the eligibility check and clicking on continue, you agree that the data of the eligibility screening can be used for study purposes.

4. **Are you 18 years or older?**
   - ❑ No *(Unfortunately, you are not eligible to participate in this study. We would like to thank you very much for your time and effort.)*
   - ❑ Yes

5. **Which of the following statements best describes your caregiving or care receiving experiences?**
   - ❑ I provide care for a family member or a friend with a chronic health condition, disability or any other care need that is 18 years old or over.
   - ❑ I receive care from a family member or a friend, that is 18 years old or older, due to my chronic health condition, disability or any other care ne
   - ❑ None of the above. *(Unfortunately, you are not eligible to participate in this study. We would like to thank you very much for your time and effort.)*

Participation in the study is entirely voluntary, but because of scientific guidelines, we need to ask you for your digital consent before taking part. To help you decide whether you want to take part in the study or not, we will first explain ho the study is conducted and what is expected from you. We kindly ask you to read this information carefully.

If you decide to participate in the study after reading the information, you can give us digital consent. This can be done online and will only take a minute. Of course, you may also decide you do not want to be part of the research. Please feel free to decide either way.
