# Supplementary material for: A Multinational Longitudinal Study Incorporating Intensive Methods to Examine Caregiver Experiences in the Context of Chronic Health Conditions: Protocol of the ENTWINE-iCohort
Source: Int J Environ Res Public Health. 2022 Jan 12;19(2):821. doi: 10.3390/ijerph19020821 (PMC8775526; doi:10.3390/ijerph19020821)
Supplement: Supplementary file 1 [file ijerph-19-00821-s001.zip › Supplementary File S2. Participant Information Sheet.pdf]

Participant Information Sheet
for
ENTWINE-iCohort Prospective Study in Informal Caregiving

You have been identified as, or identified yourself as someone who is **currently providing informal care** to a friend or loved one who has care needs arising from a health condition or because you are **currently receiving care** from a loved one due to your own health condition.

We would like to invite you to take part in a European research study. This study is led by Bangor University (UK) in collaboration with the University Medical Center of Groningen (The Netherlands), Bar-Ilan University (Israel), Uppsala University (Sweden) and the National Institute of Health and Science on Ageing (Italy). Before you decide whether to take part, it is important for you to understand why the study is being undertaken and what it involves. Please take time to read the following information carefully and discuss it with others if you wish. If there is anything that is not clear or if you would like more information, you can contact us using the details provided below.

What is the purpose of this study?

We are carrying out this large research study across the UK, the Netherlands, Italy, Israel, Sweden, Poland, Germany, Ireland and Greece in order to identify factors that are relevant and potentially important to caregivers' and care recipients' experience. By identifying the current experience and any caregiving challenges faced by a informal caregivers and care receivers in different countries with different care systems, we hope to establish who needs support, what kind, and when.
This study has five goals:

- To describe the nature and level of willingness and motivations to provide care across different countries and cultures,
- To identify factors that act as personal or geographical barriers or facilitators of caregiving,
- To explore the nature of shared experience reported by caregiver-care receiver couples/pairs,
- To identify differences across countries in the use of formal versus informal caregiving and the factors that influence this,
- To gather information about opportunities and challenges of any household-based migrant care work employed to support informal caregiving.

Do I have to take part?

Participation in this study is completely voluntary. If you decide to take part you will be asked to provide your consent in the next step on the online form you have accessed. Following consent, you are still free to withdraw at any time and without giving a reason.

What will I be asked to do if I decide to take part?

If you decide to take part, you will first complete the attached Consent Form.

You will then receive two different e-mails: one with a copy of this information letter and your consent and another one with the link to the survey to access on your smartphone, tablet or computer.

You will be asked to complete a baseline questionnaire as either a caregiver or a care recipient. Six months later you will receive a link via email inviting you to complete a follow-up questionnaire so that we can gain more information about how your situation may have changed over the last 6 months.

During the six months, we will also invite you to answer a number of short questions each week. After completing the baseline questionnaire, you will receive further instructions on how to fill in the weekly questions which can also be completed electronically on your personal device (e.g., smartphone, tablet, computer). You would receive the link by email every week for 6 months with a reminder.

If you are caring for your loved one, we would be asking for approximately sixty minutes of your time to complete the baseline and the follow-up questionnaire. Filling in the weekly questions takes about ten minutes per week.

If you receive care from your loved one, would be asking for approximately forty minutes of your time to complete the baseline and the follow-up questionnaire. Filling in the weekly questions takes about 10 minutes per week.

We would appreciate if you were able to do all parts of our study but as detailed below you have the right to complete only those questions that you wish to.

Will my taking part in this study be kept confidential?

All information collected in this study will be kept strictly confidential. Only members of the research team will have access to it. All collected data is anonymised and stored securely according to national data protection regulations. We will also follow all privacy rules in reporting of the study - no names or details that would identify specific individuals will be included. Securely stored personal data (email) will be stored for 3 years; anonymized research data will be stored for 15 years in case we need to check it and for future analyses.

You can find out more about how we use your information:

- by sending an email to our research team (entwinestudy@bangor.ac.uk)
- or by contacting Bangor University Data Protection Officer – Jenny Amphaeris (j.amphaeris@bangor.ac.uk)

In exceptional cases when information given suggests that yourself or another person is at serious risk (of severe personal distress, abuse, malpractice), the Researcher will seek your consent to share this information

with the Principal Investigator and other relevant professionals as appropriate. If your consent is not given, only in these exceptional and specific circumstances does the Researcher have the right to inform the Principal Investigator and other relevant professionals. Your agreement to this is given by initialling the box on the Consent Form.

Are there any possible disadvantages, advantages or benefits if I take part?

The information you provide in this survey will help increase understanding of the challenges and experiences of informal caregivers and care recipients. The data gathered will be useful in developing evidence-based policy recommendations and for the development of new interventions to support caregiving and care receiving. Whilst there may be no direct benefits to you, there are potential long-term benefits to others.

We know of no disadvantages or risks associated with taking part in the study, other than that we ask for some of your time. In the unlikely event that the questions in the survey cause you distress at the time of taking part, please feel that you can take a break before deciding whether or not to continue or to omit some answers. You should also keep in mind your right to withdraw from the study without providing us with a reason.

If the issues addressed raise any later questions with you, you can also contact a member of your healthcare team, your GP, or if you prefer, one of the following help-lines and organizations for support and useful information:
https://www.bangor.ac.uk/studentservices/mentalhealth/support-information.php.en#tab2

Will I get any expenses or payments if I take part?

We are unable to pay people for taking part in this study.

What will happen to the results of the study?

The results will be written up for publication in scientific and care-related journals and will likely also be presented at local, national and international conferences involving a range of audiences- academic, policy and practitioner groups, caregiving or patient organisations. In this way, it is intended that our findings inform future research, policy and practice to benefit support for caregiving and care receiving. You will also be asked if you would like a copy of the study findings.

Who is organising this study?

This study is being carried out by Mikołaj Zarzycki, Eva Bei, Giulia Ferraris, Saif Elayan and Oliver Fisher as part of their PhD research within a large EU funded ENTWINE project supporting research in informal caregiving. It is supervised by Prof Val Morrison (Principal Investigator at Bangor University), Dr Noa Vilchinsky (Bar-Ilan University), Prof Mariët Hagedoorn and Prof Erik Buskens (University of Groningen, University Medical Center Groningen) and Dr Giovanni Lamura (National Institute of Health and Science on Ageing, Italy).

Who can I contact for further information?

For more information about this research, please contact:

ENTWINE-iCohort Study Research Team
Email address: entwinestudy@bangor.ac.uk

Principal Investigator of the Study
Prof Val Morrison
School of Psychology
Brigantia Building
Bangor University
Penrallt Rd, Bangor LL57 2AS

Telephone for the UK, Ireland and Poland: 01248383010

Who has reviewed the ethics of this study?

Full approvals have been obtained from: Bangor University, School of Psychology Research Ethics and Governance Committee and the NHS Research Ethics Committee, Ref Number 20/WA/0006.

What happens after the study?

If you would like to receive a summary of the findings once the study has been completed, please simply initial the relevant box on the Consent Form.

Complaints:

If you have any complaints about the way this research has been conducted, please contact:

For UK completers: Mr Huw Ellis, Manager, School of Psychology, Bangor University, Bangor, Gwynedd, LL57 2AS; email address: h.ellis@bangor.ac.uk

Thank you for taking the time to read this Information Sheet.
