# Supplementary material for: A Multinational Longitudinal Study Incorporating Intensive Methods to Examine Caregiver Experiences in the Context of Chronic Health Conditions: Protocol of the ENTWINE-iCohort
Source: Int J Environ Res Public Health. 2022 Jan 12;19(2):821. doi: 10.3390/ijerph19020821 (PMC8775526; doi:10.3390/ijerph19020821)
Supplement: Supplementary file 1 [file ijerph-19-00821-s001.zip › Supplementary File S3. Participant Consent Forms to participate in the survey.pdf]

Participant Consent Form

Title of Project: ENTWINE Prospective Cohort Study in Informal Care (ENTWINE-iCohort)

Names of Researchers: Mikołaj Zarzycki, Eva Bei, Giulia Ferraris, Saif Elayan and Oliver Fisher

By signing this consent form, you agree to the following statements:

1. I confirm that I have read *Participant Information Sheet for the ENTWINE iCohort Prospective Study in Informal Caregiving* for the above study. I have had the opportunity to consider the information, ask questions and have had these answered satisfactorily.

2. I understand that my participation is voluntary and that I am free to withdraw at any time without giving any reason and without my medical care or legal rights being affected.

3. I understand that all information collected in this study will be kept strictly confidential. Only members of the research team will have access to your data which will be anonymised and stored securely. We will also follow all privacy rules in reporting the study - no identifying details of specific individuals will be included. Securely stored personal data (email) will be stored for 3 years and anonymized research data will be stored for 15 years.

☑ I have read the above information, and I agreed to take part in the above study.

This form will be produced in duplicate. One digital copy should be retained by the participant and the other will be sent electronically to the ENTWINE iCohort management team.

Participant Consent Form for the Follow-up Survey

Title of Project: ENTWINE Prospective Cohort Study in Informal Care (ENTWINE-iCohort)

Names of Researchers: Mikołaj Zarzycki, Eva Bei, Giulia Ferraris, Saif Elayan and Oliver Fisher

By signing this consent form, you agree to the following statements:

1. I confirm that I have read *Participant Information Sheet for the ENTWINE iCohort Prospective Study in Informal Caregiving* for the above study. I have had the opportunity to consider the information, ask questions and have had these answered satisfactorily.

2. I understand that my participation is voluntary and that I am free to withdraw at any time without giving any reason and without my medical care or legal rights being affected.

3. I understand that all information collected in this study will be kept strictly confidential. Only members of the research team will have access to it. Everyone involved in this study will keep your data safe and secure. We will also follow all privacy rules. No names or details that would identify specific individuals will be included in study reports. At the end of the study we will save some of the data in case we need to check it and for future research. Securely stored personal data (email) will be stored for 3 years; anonymized research data will be stored for 15 years.

☑ I have read the above information, and I agreed to take part in the above study.

This form will be produced in duplicate. One digital copy should be retained by the participant and the other will be sent electronically to the ENTWINE iCohort management team.
