# Supplementary material for: A Multinational Longitudinal Study Incorporating Intensive Methods to Examine Caregiver Experiences in the Context of Chronic Health Conditions: Protocol of the ENTWINE-iCohort
Source: Int J Environ Res Public Health. 2022 Jan 12;19(2):821. doi: 10.3390/ijerph19020821 (PMC8775526; doi:10.3390/ijerph19020821)
Supplement: Supplementary file 1 [file ijerph-19-00821-s001.zip › Supplementary File S6. ENTWINE-iCOHORT Caregiver Follow up Survey.pdf]

ENTWINE

INFORMAL CARE

The European Training Network on Informal Care

ENTWINE-iCOHORT CAREGIVER FOLLOW-UP SURVEY

This study is being carried out by Bangor University (UK), University Medical Center of Groningen (The Netherlands), National Institute for Health and Science on Ageing (Italy), Bar-Ilan University (Israel) and Uppsala University (Sweden).

About six months ago you filled in a survey relating to your experiences in caregiving. You may notice that many of the questions in this survey are the same as the questions in the initial questionnaire. We ask the same questions, as by monitoring any changes in your caregiving experiences and the experiences of your loved one, we can create a more nuanced understanding of the long term caring needs and challenges of informal caregivers. This will be crucial in developing new interventions and policies to support informal caregivers.

Instructions for this questionnaire:

- If you read 'your loved one', then the question refers to the person for whom you are a caregiver. (the same person you referred to in the first questionnaire approximately six months ago).
- Read each question through completely before answering.
- An "Other" option is provided in case you believe that the response options do not describe your situation or the situation of your loved one adequately.
- If you need to take breaks while completing the survey, you can close the survey window at any point and your responses will be automatically saved. We recommend that you fill in this questionnaire using a laptop or tablet.

1. **Please select from the list below your current country of residence.**
   - ❑ UK
   - ❑ the Netherlands
   - ❑ Greece
   - ❑ Italy
   - ❑ Ireland
   - ❑ Poland
   - ❑ Sweden
   - ❑ Germany
   - ❑ Israel
   - ❑ Other (specify): _______________

2. **Are you still providing care to the same person you referred to in the first questionnaire approximately six months ago?**
   - ❑ No
   - ❑ Yes (skip to question 9)

3. **Why are you no longer providing care to your loved one?**
   - ❑ My loved one died
   - ❑ My loved one is being for by another caregiver
   - ❑ My loved one no longer needs care
   - ❑ My loved one has entered formal residential care
   - ❑ Other (specify): __________________
   - ❑ Prefer not to say

4. **In the future, how willing would you be to take on a caregiving role again?**

- ❑ Completely unwilling
- ❑ Somewhat unwilling
- ❑ Not sure
- ❑ Somewhat willing
- ❑ Completely willing

5. **The World Health Organisation-Five Well-Being Index (WHO-5)**

	Over the last two weeks	All of the time	Most of the time	More than half of the time	Less than half of the time	Some of the time	At no time
**1**	I have felt cheerful and in good spirits	5	4	3	2	1	0
**2**	I have felt calm and relaxed	5	4	3	2	1	0
**3**	I have felt active and vigorous	5	4	3	2	1	0
**4**	I woke up feeling fresh and rested	5	4	3	2	1	0
**5**	My daily life has been filled with things that interest me	5	4	3	2	1	0

6. **Meaning in Life Questionnaire (MLQ)** (Steger et al., 2006)

Please take a moment to think about what makes your life and existence feel important and significant to you. Please respond to the following statements as truthfully and accurately as you can, and also please remember that these are very subjective questions and that there are no right or wrong answers. Please answer according to the scale below:

	**Absolutely untrue**	**Mostly Untrue**	**Somewhat Untrue**	**Can't Say True or False**	**Somewhat True**	**Mostly True**	**Absolutely True**
I understand my life's meaning.							

I am looking for something that makes my life feel meaningful.							
I am always looking to find my life's purpose.							
My life has a clear sense of purpose.							
I have a good sense of what makes my life meaningful.							
I have discovered a satisfying life purpose.							
I am always searching for something that makes my life feel significant.							
I am seeking a purpose or mission for my life.							

My life has no clear purpose.							
I am searching for meaning in my life.							

7. **10-item Center for Epidemiologic Studies Depression Scale (CESD-10)** (Andersen et al., 1994)

Below is a list of the ways you might have felt or behaved. Please indicate how often you have felt this way during the past week.

	**Rarely or none of the time (less than 1 day)**	**Some or a little of the time (1-2 days)**	**Occasionally or a moderate amount of time (3-4 days)**	**All of the time (5-7 days)**
I was bothered by things that usually don't bother me				
I had trouble keeping my mind on what I was doing				
I felt depressed				
I felt that everything I did was an effort				
I felt hopeful about the future				
I felt fearful				
My sleep was restless				
I was happy				
I felt lonely				
I could not "get going"				

Please answer question number 8 only if you replied "my loved one died" to question number 3

8. **To help you deal with feelings of loss, grief, loneliness or missing your loved one, which of the following actions have you taken?**
   Please select all that apply

   - ❑ Gone to a self-help group or support group for people who have lost a loved one
   - ❑ Spoken to a religious leader
   - ❑ Talked to your family doctor
   - ❑ Gone to a mental health professional for help—someone like a social worker or psychologist
   - ❑ Other (specify): _____________
   - ❑ None of the above

Thank you for completing our survey.

We very much appreciate the information that you have provided and your time taken in completing this survey. You can find the latest updates on our project at: https://www.entwine-icohort.eu/

If you have any further questions on the survey, please send us an email at: entwinestudy@bangor.ac.uk

9. **Has any of the following changed since you completed the last survey approximately six months ago?**

Please answer "yes" or "no" to each item below.	**No**	**Yes**
Your address		
Your marital/relationship status		
The caregiving benefits you receive, if any. (e.g., cash benefits, care-leave benefits, tax benefits, coverage of social or pension contributions)		
Your own health condition		
Your work/employment situation (i.e. stopped or started working, change in number of working hours, change in income from employment, etc...		

10. **Since the last assessment, would you say your loved one's health condition has:**

    - ❑ stayed the same
    - ❑ significantly worsened
    - ❑ significantly improved

11. **What is your gender?**
    - ❑ Female
    - ❑ Male
    - ❑ Non-binary / third gender
    - ❑ Prefer to self describe:
    - ❑ Prefer not to say

12. **Which city/town do you live in?**

____

13. **Is your home located in?**
    - ❑ central city/area
    - ❑ peripheral area in the city
    - ❑ rural area

14. **Please indicate the highest level of education you have achieved**
    - ❑ Primary
    - ❑ Secondary
    - ❑ Post-secondary vocational education (e.g., further education college, apprenticeships etc.)
    - ❑ Post secondary academic education (e.g., university)
    - ❑ Not listed or other

15. **What is your marital status?**
    - ❑ Single (never married) (skip to question 17)
    - ❑ Married
    - ❑ In a civil union/domestic partnership
    - ❑ Divorced (skip to question 17)
    - ❑ Widowed (skip to question 17)
    - ❑ Other (specify) _______________

16. **For how long have you been in this relationship?**

    Years _________   Months________

17. **Do you have children?**

    If you answer "yes", please specify how many children you have.

    - ❑ No (skip to question 20)
    - ❑ Yes. I have _____child(ren)

18. **Do you have children under 18 years of age?**

    If you answer "yes", please specify how many children under 18 years old you have.

    ❑ No
    ❑ Yes. I have _______ children under 18 years of age.

19. **Do your children live in your home?**

    If you answer "yes", please specify how many children live in your home.

    ❑ No
    ❑ Yes. _____child(ren) live in my home

Skip to question 26 if you answered "no" to "Your work/employment situation (i.e. stopped or started working, change in number of working hours, change in income from employment, etc..." in question 9

20. **Are you currently employed or self-employed (working for pay)?**
    ❑ No
    ❑ Yes

21. **During the COVID-19 pandemic have you lost your job?**
    ❑ No
    ❑ No, but I started to work from home as a result of the COVID-19 situation
    ❑ Yes, permanently
    ❑ Yes, temporarily

22. **When you compare your financial situation before COVID-19 pandemic and now would you say it has become better worse or remained the**
    ❑ Better
    ❑ The same
    ❑ Worse
    ❑ Do not know

Skip to question 26 if you answered "no" to question 20

23. **Are you currently working full-time (e.g., according to your employment contract)?**
    ❑ No. I work part-time: _______hours per week.
    ❑ Yes, I work full time: _______hours per week.

24. After any taxes and contributions, what is your average monthly income from employment?

Please include any additional or extra or lump sum payment, such as bonuses, 13 month, Christmas or Summer pays.

- ❑ _____________ (skip to question 26)
- ❑ I prefer not to answer this question.

25. Please indicate your monthly income level after taxes and contributions.[1]

Please include any additional or extra or lump sum payment, such as bonuses, 13 month, Christmas or Summer pays.

- ❑ Less than £800
- ❑ Between £800 and 1500
- ❑ Between £1500 and £4000
- ❑ More than £4000
- ❑ Prefer not to answer this question

26. What is your religion, if any?[2]

- ❑ Protestant
- ❑ Roman Catholic
- ❑ Eastern Orthodox
- ❑ Islam
- ❑ Jewish
- ❑ Buddhist
- ❑ Hindu
- ❑ Other (specify)
- ❑ I am not religious (skip to question 28)
- ❑ Prefer not to say

27. What is the importance of religion in your life?

- ❑ Not important at all
- ❑ Not too important
- ❑ Fairly important
- ❑ Very important

28. Do you have any of the following?

Please select all that apply.

- ❑ A physical impairment or disability

---

[1] The income brackets provided in this question were applied in the UK. Income brackets for other countries were adjusted to reflect differences in average incomes of the population. These values were based on the Survey of Health, Ageing and Retirement in Europe (SHARE).

[2] Question not asked in Italy, Sweden and the Netherlands due to ethical reasons

- ❑ Sight or hearing loss
- ❑ A mental health problem or illness
- ❑ A learning disability or difficulty
- ❑ A long-standing illness
- ❑ Multimorbidity
- ❑ Other (please specify)
- ❑ None of the above

29. How many people aged 18 or over do you provide informal care for?

____

30. Have you been diagnosed with COVID-19?

- ❑ No
- ❑ Yes, but I was not admitted to a hospital or intensive care unit
- ❑ Yes, and I was admitted to a hospital but not an intensive care unit
- ❑ Yes, and I was admitted to an intensive care unit
- ❑ Prefer not to say

We will refer to the person you provide care for as "your loved one" (the same person you referred to in the first questionnaire six months ago).

31. What is the gender of your loved one?

- ❑ Female
- ❑ Male
- ❑ Non-binary / third gender
- ❑ Prefer to self describe:
- ❑ Prefer not to say

32. Has your loved one been diagnosed with COVID-19?

- ❑ No
- ❑ Yes, but they were not admitted to a hospital or intensive care unit
- ❑ Yes, and they were admitted to a hospital but not an intensive care unit
- ❑ Yes, and they were admitted to an intensive care unit
- ❑ Prefer not to say

*Skip to question 36 if you answered “*stayed the same” *to question 10*

33. What health condition(s) has your loved one been diagnosed with?

Please select all that apply.

- ❑ A heart attack including myocardial infarction or coronary thrombosis or any other heart problem including congestive heart failure
- ❑ High blood pressure or hypertension
- ❑ High blood cholesterol

- ❑ A stroke or cerebral vascular disease
- ❑ Diabetes or high blood sugar
- ❑ Chronic lung disease such as chronic bronchitis or emphysema
- ❑ Cancer (please specify the type of cancer if known).
- ❑ Stomach or duodenal ulcer, peptic ulcer
- ❑ Parkinson disease
- ❑ Cataracts
- ❑ Hip fracture
- ❑ Other fractures
- ❑ Alzheimer's disease, dementia, organic brain syndrome, senility or any other serious memory impairment, please specify:
- ❑ Multiple sclerosis
- ❑ Rheumatoid Arthritis
- ❑ Osteoarthritis, or other rheumatism
- ❑ Chronic kidney disease
- ❑ Traumatic brain injury
- ❑ HIV/AIDS
- ❑ Other condition(s) _________
- ❑ They have not been diagnosed with any conditions (skip to question 37)

34. Which one of these health conditions do you think causes the most limitations to your loved one?

____

35. How long has your loved one been living with this health condition?

Years ______ Months_____

36. Do you share a household with your loved one?

- ❑ No
- ❑ Yes (skip to question 43)

37. Where does your loved one live?

- ❑ In their own home
- ❑ In someone else's home
- ❑ In an assisted living facility or other residential setting where some care is provided
- ❑ In a nursing care or long-term care facility (skip to question 39)

38. Indicate the option that best describes your loved one's living arrangement:

Please select all that apply.

- ❑ Alone
- ❑ With their children

- ❑ With their partner
- ❑ With paid carers
- ❑ With others, please specify

39. Which city/town does your loved one live in?

____

40. Is the place where your loved one lives, located in a:

- ❑ central city/area
- ❑ peripheral area in the city
- ❑ rural area

41. How do you reach the place where your loved one lives?

- ❑ I walk to their home
- ❑ I cycle to their home
- ❑ I drive to their home
- ❑ I use public transportation (bus or train)
- ❑ If Other, please specify

42. How far is your home from the place where your loved one lives?

- ❑ less than 15 minutes
- ❑ 15 to 30 minutes
- ❑ 30 to 60 minutes
- ❑ 60 to 90 minutes
- ❑ 90 to 120 minutes
- ❑ More than 120 minutes

43. Inclusion of Other in the Self Scale (IOS) (Aron et al., 1992)

Below there are seven pairs of circles: the level of overlap between the circles indicates how close you feel to your loved one. The greater the overlap, the closer you feel to your loved one. Please indicate the picture that best describes your relationship with your loved one.

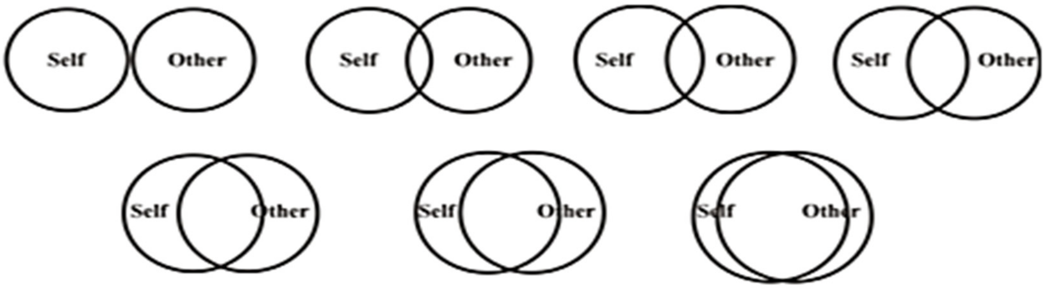

44. **Does your loved one have any difficulty with these activities?** (Katz, 1983)

Please select all that apply.

- ❑ Dressing, including putting on shoes and socks
- ❑ Walking across a room
- ❑ Bathing or showering
- ❑ Eating, such as cutting up their food
- ❑ Getting in or out of bed
- ❑ Using the toilet, including getting up or down
- ❑ None of these

Please fill in the following questions in relation to the **last week OR on a typical week** if last week was a holiday, or if you were away, or ill etc)

A limit of 126 hours has been set for the number of hours of care that you provide each week. The purpose of this limit is not to undervalue or underestimate the care that you provide. This limit has been set to allow you time (6 hours per day) for basic needs such as resting/sleeping and doing non-care related tasks.

45. **During the last week, on how many days did you provide care to your loved one?**

- ❑ 0 days
- ❑ 1 day
- ❑ 2 days
- ❑ 3 days
- ❑ 4 days
- ❑ 5 days
- ❑ 6 days
- ❑ 7 days

46. **During the last week, how much time did you spend on household activities and tasks that you would not have had to perform if your loved one was in good health, or if they could have done them independently?**

For example preparing food, cleaning, washing, ironing, sewing, shopping, and gardening.

______hours per week.

47. **During the last week, how much time did you spend helping your loved one with their personal care?**

For example dressing/undressing, washing/showering/bathing, hair care, shaving and grooming, and going to the toilet.

_________hours per week.

48. **During the last week, how much time did you spend providing practical support to your loved one that would not have had to be performed if they were in good health, or if they could have done it independently?**

For example eating and drinking, moving inside or outside the house (including assistance with walking or using a wheelchair), visiting family or friends, accompany to healthcare visits (e.g. doctor appointments), filling prescriptions at the pharmacy, help taking medications, and taking care of financial matters (e.g. paying the bills or managing healthcare insurance).

_____ hours per week.

49. **During the last week, how much time did you spend on providing emotional support to your loved one that would not have had to be provided**

For example help to cope with pain, disability, and discomfort, anxiety, worry, and loneliness.

_____hours per week.

50. **Due to COVID-19 the hours of emotional support you provide have:**
    - ❑ Increased by ______ hour(s) per week
    - ❑ Decreased by ____ hour(s) per week
    - ❑ Stayed the same

51. **Due to COVID-19 the hours of practical support you provide have:**
    - ❑ Increased by ____ hour(s) per week
    - ❑ Decreased by ____ hour(s) per week
    - ❑ Stayed the same

52. **Due to COVID-19 the hours of personal care you provide have:**
    - ❑ Increased by hour(s) per week
    - ❑ Decreased by hour(s) per week
    - ❑ Stayed the same

53. **Besides your care or support, does she/he also receive care from other informal caregivers?**
    - ❑ No, I am the only informal caregiver (skip to question 57)
    - ❑ Yes, from __________________________________ [number] other informal caregivers

54. **In the past week, how many hours of care did these informal caregiver(s) provide for your loved one?**
    - ❑ They have not provided any care.
    - ❑ They have provided hours of care.
    - ❑ Do not know

55. **Do you consider yourself to be the person who provides most of the informal care for your loved one?**

- ❑ No
- ❑ Yes (skip to question 57)

56. Who would you consider to be the person who provides most of the informal care for your loved one? Their:

- ❑ Spouse / Partner
- ❑ Child
- ❑ Parent
- ❑ Sibling
- ❑ Daughter- / Son-in-law
- ❑ Uncle / Aunt
- ❑ Nephew/ Niece
- ❑ Cousin
- ❑ Friend
- ❑ Neighbour
- ❑ Other, please specify

57. Skip to question 68 if you answered "In a nursing care or long-term care facility" to question 37

Now we want to ask you about the services that your loved one may have received from paid care worker(s), sitting services and overnight care.

Over the past three months, has your loved one been provided with home care also by one or more paid care workers?

- ❑ No (skip to question 68)
- ❑ Yes

58. How many paid care workers are involved with your loved one's home care?

- ❑ 1
- ❑ 2 (skip to question 64)
- ❑ 3 (skip to question 64)
- ❑ 4 (skip to question 64)
- ❑ More than 4 (skip to question 64)

59. Was the paid worker a migrant care worker?

"Migrant worker" refers to a person who is employed in a country that they migrated to. This includes not only people who migrated for the purpose of employment, but also those who migrated for other reasons (eg., family or education) and then gained employment at a later date.

- ❑ No (skip to question 62)
- ❑ Yes
- ❑ Do not know (skip to question 62)

60. **Please fill in the follow table based on the situation of the migrant care worker**

**Employed on a live-in basis?**	**Gender**	**Age**	**Level of education**	**Marital status**
No	Female	18-24	Primary	Single (never married)
Yes	Male	25-34	Secondary	Married
	Non-binary /third gender	35-44	Post-secondary vocational education (e.g., further education college, apprenticeships etc.)	In a civil union/domestic partnership
	Do not know	45-54	Post secondary academic education (e.g., university)	Divorced
		55-64	Not listed or other	Widowed
		65-74	Do not know	Do not know
		75+		
		Do not know		

61. **What is the nationality of the migrant care worker?**

They are a national/citizen of:

____

Skip to question 68 if you answered "yes" to question 59

62. **Please fill in the follow table based on the situation of the paid care worker**

**Employed on a live-in basis?**	**Gender**	**Age**	**Level of education**	**Marital status**
No	Female	18-24	Primary	Single (never married)

Yes	Male	25-34	Secondary	Married
	Non-binary /third gender	35-44	Post-secondary vocational education (e.g., further education college, apprenticeships etc.)	In a civil union/domestic partnership
	Do not know	45-54	Post secondary academic education (e.g., university)	Divorced
		55-64	Not listed or other	Widowed
		65-74	Do not know	Do not know
		75+		
		Do not know		

63. Does the paid care worker have a migration background?

"Migration background" refers to a person who has:

(a) migrated into their present country of residence; and/or

(b) previously had a different nationality from their present country of residence; and / or

(c) at least one of their parents previously entered their present country of residence as a migrant.

- ❑ No
- ❑ Yes
- ❑ Do not know

Skip to question 68 if you answered "no" or "do not know" to question 59

64. Were any of these paid workers a migrant worker?

“Migrant worker" refers to a person who is employed in a country that they migrated to. This includes not only people who migrated for the purpose of employment, but also those who migrated for other reasons (eg., family or education) and then gained employment at a later date.

- ❑ No (skip to question 68)
- ❑ Yes
- ❑ Do not know (skip to question 68)

65. Please fill in a separate row in the table for each individual worker. "Migration background" refers to a person who has:

(a) migrated into their present country of residence; and/or

(b) previously had a different nationality from their present country of residence; and / or

(c) at least one of their parents previously entered their present country of residence as a migrant.

	**Employed on a live-in basis?**	**Migrant worker?**	**Migration background ?**	**Gender**	**Age**	**Level of education**	**Marital status**
Care worker n. 1	No<br><br>Yes	No<br><br>Yes<br><br>Do not know	No<br><br>Yes<br><br>Do not know	Female<br><br>Male<br><br>Non-binary /third gender<br><br>Do not know	18-24<br><br>25-34<br><br>35-44<br><br>45-54<br><br>55-64<br><br>65-74<br><br>75+<br><br>Do not know	Primary<br><br>Secondary<br><br>Post-secondary vocational education (e.g., further education college, apprenticeships etc.)<br><br>Post secondary academic education (e.g., university)<br><br>Not listed or other	Single (never married)<br><br>Married<br><br>In a civil union/domestic partnership<br><br>Divorced<br><br>Widowed<br><br>Do not know

						Do not know	
Care worker n. 2	No<br><br>Yes	No<br><br>Yes<br><br>Do not know	No<br><br>Yes<br><br>Do not know	Female<br><br>Male<br><br>Non-binary /third gender<br><br>Do not know	18-24<br><br>25-34<br><br>35-44<br><br>45-54<br><br>55-64<br><br>65-74<br><br>75+<br><br>Do not know	Primary<br>Secondary<br>Post-secondary vocational education (e.g., further education college, apprenticeships etc.)<br>Post secondary academic education (e.g., university)<br>Not listed or other<br>Do not know	Single (never married)<br><br>Married<br><br>In a civil union/domestic partnership<br><br>Divorced<br><br>Widowed<br><br>Do not know
Care worker n. 3	No<br><br>Yes	No<br><br>Yes<br><br>Do not know	No<br><br>Yes<br><br>Do not know	Female<br><br>Male<br><br>Non-binary /third gender<br><br>Do not know	18-24<br>25-34<br>35-44<br>45-54<br>55-64<br>65-74<br>75+<br>Do not know	Primary<br><br>Secondary<br><br>Post-secondary vocational education (e.g., further education college, apprenticeships etc.)<br><br>Post secondary academic education (e.g., university)<br><br>Not listed or other<br><br>Do not know	Single (never married)<br><br>Married<br><br>In a civil union/domestic partnership<br><br>Divorced<br><br>Widowed<br><br>Do not know

Care worker n. 4	No<br><br>Yes	No<br><br>Yes<br><br>Do not know	No<br><br>Yes<br><br>Do not know	Female<br><br>Male<br><br>Non-binary /third gender<br><br>Do not know	18-24<br><br>25-34<br><br>35-44<br><br>45-54<br><br>55-64<br><br>65-74<br><br>75+<br><br>Do not know	Primary<br><br>Secondary<br><br>Post-secondary vocational education (e.g., further education college, apprenticeships etc.)<br><br>Post secondary academic education (e.g., university)<br><br>Not listed or other<br><br>Do not know	Single (never married)<br><br>Married<br><br>In a civil union/domestic partnership<br><br>Divorced<br><br>Widowed<br><br>Do not know

66. What is the nationality of the migrant care worker(s)?

They are a national/citizen of:

Care worker n. 1 _________

Care worker n. 2 _________

Care worker n. 3 _________

Care worker n. 4 _________

Skip to question 68 if you answered "yes" to question 64

67. Please fill in a separate row in the table for each individual worker. "Migration background" refers to a person who has:

(a) migrated into their present country of residence; and/or

(b) previously had a different nationality from their present country of residence; and / or

(c) at least one of their parents previously entered their present country of residence as a migrant.

	**Employed on a live-in basis?**	**Migration background?**	**Gender**	**Age**	**Level of education**	**Marital status**
Care worker n. 1	No<br>Yes	No<br>Yes<br>Do not know	Female<br>Male<br>Non-binary /third gender<br>Do not know	18-24<br>25-34<br>35-44<br>45-54<br>55-64<br>65-74<br>75+<br>Do not know	Primary<br>Secondary<br>Post-secondary vocational education (e.g., further education college, apprenticeships etc.)<br>Post secondary academic education (e.g., university)<br>Not listed or other<br>Do not know	Single (never married)<br>Married<br>In a civil union/domestic partnership<br>Divorced<br>Widowed<br>Do not know
Care worker n. 2	No<br>Yes	No<br>Yes<br>Do not know	Female<br>Male<br>Non-binary /third gender<br>Do not know	18-24<br>25-34<br>35-44<br>45-54<br>55-64<br>65-74	Primary<br>Secondary<br>Post-secondary vocational education (e.g., further education college, apprenticeships etc.)<br>Post secondary	Single (never married)<br>Married<br>In a civil union/domestic partnership

				75+<br><br>Do not know	academic education (e.g., university)<br>Not listed or other<br>Do not know	Divorced<br><br>Widowed<br><br>Do not know
Care worker n. 3	No<br><br>Yes	No<br><br>Yes<br><br>Do not know	Female<br><br>Male<br><br>Non-binary /third gender<br><br>Do not know	18-24<br>25-34<br>35-44<br>45-54<br>55-64<br>65-74<br>75+<br>Do not know	Primary<br><br>Secondary<br><br>Post-secondary vocational education (e.g., further education college, apprenticeships etc.)<br><br>Post secondary academic education (e.g., university)<br><br>Not listed or other<br><br>Do not know	Single (never married)<br><br>Married<br><br>In a civil union/domestic partnership<br><br>Divorced<br><br>Widowed<br><br>Do not know
Care worker n. 4	No<br><br>Yes	No<br><br>Yes<br><br>Do not know	Female<br><br>Male<br><br>Non-binary /third gender<br><br>Do not know	18-24<br><br>25-34<br><br>35-44<br><br>45-54<br><br>55-64<br><br>65-74	Primary<br><br>Secondary<br><br>Post-secondary vocational education (e.g., further education college,	Single (never married)<br><br>Married<br><br>In a civil union/domestic partnership

				75+<br><br>Do not know	apprentices hips etc.)<br><br>Post secondary academic education (e.g., university)<br><br>Not listed or other<br><br>Do not know	Divorced<br><br>Widowed<br><br>Do not know

68. Besides your care or support, does your loved one visit a daycare facility or a residential or nursing home?

- ❑ No
- ❑ Yes, for ___ hour(s) during the last week.

The next questions will ask about the services that you, the caregiver, may have used.

69. Have you received any of the following services in the past six months?

- ❑ Information about the disease that the loved one has.
- ❑ Coordination services providing information and advice about the type of help, support and benefits that is available to carers and how to access it (e.g. information centers, case (or care) managers, advice or guidance from a physician or social worker.
- ❑ Training to help carers develop the skills they need to care and cope with the burden of care
- ❑ Counselling (i.e. supportive and social counselling, etc.).
- ❑ Self-help or support group
- ❑ Formal standardized assessment of your needs or caring situation
- ❑ Reconciliation of caring and employment (e.g. flexible work arrangements and paid and/or unpaid care leaves from work).
- ❑ In-home respite care (temporary or short-term care provided at home to give rest or relief to informal caregivers from the tasks of caregiving).
- ❑ Out-of-home respite care (e.g. day-care services and temporary stay at a residential or nursing home).
- ❑ None of them (skip to question 74)

For question 70 and 71, only complete the options that correspond to the options you selected in question 69.

70. In a typical month, how many times do you use the following service(s)?

- ❑ Information about the disease that the loved one has _______________

- ❑ Coordination services providing information and advice about the type of help, support and benefits that is available to carers and how to access it (e.g. information centers, case (or care) managers, advice or guidance from a physician or social worker) _______________

- ❑ Training to help carers develop the skills they need to care and cope with the burden of care. _______________

- ❑ Counselling (i.e. supportive and social counselling, etc.) _______________

- ❑ Self-help or support group _______________

- ❑ In-home respite care (temporary or short-term care provided at home to give rest or relief to informal caregivers from the tasks of caregiving) _______________

- ❑ Out-of-home respite care (e.g. day-care services and temporary stay at a residential or nursing home). _______________

71. Which of these services have you received online?

Please select all that apply.

- ❑ Information about the disease that the loved one has.
- ❑ Coordination services providing information and advice about the type of help, support and benefits that is available to carers and how to access it (e.g. information centers, case (or care) managers, advice or guidance from a physician or social worker).
- ❑ Training to help carers develop the skills they need to care and cope with the burden of care.
- ❑ Counselling (i.e. supportive and social counselling, etc.).
- ❑ Self-help or support group.
- ❑ None

72. Given the COVID-19 situation, do you still have access to the usual support services you receive as a caregiver?

- ❑ No (skip to question 74)
- ❑ Yes (skip to question 74)
- ❑ Yes, but I have to receive some of these services online due to COVID-19

73. Please indicate which of the following services you receive online due to COVID-19:

Please select all that apply.

- ❑ Information about the disease that the loved one has.
- ❑ Coordination services providing information and advice about the type of help, support and benefits that is available to carers and how to access it (e.g. information centers, case (or care) managers, advice or guidance from a physician or social worker).
- ❑ Training to help carers develop the skills they need to care and cope with the burden of care.
- ❑ Counselling (i.e. supportive and social counselling, etc.).
- ❑ Self-help or support group.
- ❑ If Other please specify _____

74. *Skip to question 75 if you selected no to "The caregiving benefits you receive, if any. (e.g., cash benefits, care-leave benefits, tax benefits, coverage of social or pension contributions) " in question 9*

Do you receive any of the following benefits because of your caregiving role?

- **Cash benefits (e.g. carer allowance, carer's benefit, care wage, cash for care, paid kin caregiver, bonuses, etc)**

  - ❑ No
  - ❑ Yes. I receive ____ per month.

- **Financial compensation during care leave (e.g. care-leave benefits, career break compensation, family hospice leave grant, income replace**

  - ❑ No
  - ❑ Yes. I receive _____ per month.

- **Tax benefits such as exemptions, deductions, and credits (e.g. council tax reduction, deductions from income tax, etc.)**

  - ❑ No
  - ❑ Yes

- **Coverage of social or pension contributions (e.g. contributions are wholly or partly paid by the government or insurance).**

  - ❑ No
  - ❑ Yes

- **Carer/caregiver credits (i.e. pension credits received for providing care).**

- ❑ No
- ❑ Yes

- **Health insurance**
  - ❑ No
  - ❑ Yes

75. Motivations in Elder Care Scale (MECS) (Lyonette & Yardley, 2003)

The following statements describe some of the reasons why people begin caring for a relative or friend. Please answer each question by choosing the option which best describes your own situation.

	Strongly disagree	Disagree	Neither agree or disagree	Agree	Strongly agree
I felt that I had no choice but to care for my loved one:					
I would feel guilty if I didn't care for the loved one:					
The loved one expected me to care for them:					
I felt that people would disapprove if I didn't care for the loved one:					
It's part of my nature to care for others:					
I felt it was my duty to care for the loved one:					
I wanted to make sure the loved one was safe:					
Caring for the loved one was an automatic decision:					
I do not/did not want the loved one to go into a home:					
I wanted to provide care for the loved one myself:					
Caring for the loved one is a way of living up to my principles:					
The loved one was gradually becoming more dependent on me:					

I felt that I had a responsibility towards the loved one:					

76. **Partner-Specific Communal Motivation scale (CMS)** (Lemay & Neal, 2013)

Keeping in mind your loved one, indicate the extent to which each statement applies to you.

	Not at all								Extremely
Helping my loved one is a high priority for me.	1	2	3	4	5	6	7	8	9
I could easily put my loved one's needs out of my thoughts.	1	2	3	4	5	6	7	8	9
I care for my loved one.	1	2	3	4	5	6	7	8	9
I care about my loved one's well-being.	1	2	3	4	5	6	7	8	9
I could easily accept not helping my loved one.	1	2	3	4	5	6	7	8	9
I would sacrifice very much to help my loved one.	1	2	3	4	5	6	7	8	9
I would incur a large cost in order to help my loved one.	1	2	3	4	5	6	7	8	9
I care for my loved one's needs.	1	2	3	4	5	6	7	8	9
I would go out of my way to help my loved one.	1	2	3	4	5	6	7	8	9
I would be reluctant to sacrifice for my loved one.	1	2	3	4	5	6	7	8	9

77. **Willingness to care scale (WTC)** (Abell, 2001)

Caregiving can be a demanding and sometimes overwhelming experience. Caregivers may differ in the tasks they feel able and/or willing to perform.

Being able to perform a task means that you believe you could do it if necessary. Being willing to perform a task means that you feel you would do it if it had to be done.

For example: I am able to change soiled sheets but I would not be completely willing to do so.

As you read the statements below, think about your loved one.

First, choose yes for each one of the tasks you feel able to do for your loved one. Second, reread the items, and choose the option which best shows how willing you are to do each one.

	Able? (No/Yes)	How willing?

Listen to your loved one when they are sad.		1	2	3	4	5
Comfort your loved one when they are upset.		1	2	3	4	5
Help your loved one deal with anxiety about the future.		1	2	3	4	5
Hold hands when your loved one is afraid.		1	2	3	4	5
Encourage your loved one when they feel hopeless.		1	2	3	4	5
Listen to your loved one's concerns about death or dying.		1	2	3	4	5
Help keep your loved one's spirits up.		1	2	3	4	5
Hold your loved one when they are crying.		1	2	3	4	5
Listen to your loved one when they are angry.		1	2	3	4	5
Be patient when your loved one is disoriented or confused.		1	2	3	4	5
Take your loved one to a medical appointment.		1	2	3	4	5
Bring home the groceries for your loved one.		1	2	3	4	5
Help pay for your loved one's medicine.		1	2	3	4	5
Prepare meals for your loved one.		1	2	3	4	5
Clean your loved one's room or home.		1	2	3	4	5
Wash your loved one's dishes.		1	2	3	4	5
Do your loved one's laundry.		1	2	3	4	5
Help pay for your loved one's food or housing.		1	2	3	4	5
Have your loved one live in your home.		1	2	3	4	5
Negotiate your loved one's health care options with a doctor.		1	2	3	4	5
Help your loved one take medicine.		1	2	3	4	5
Change your loved one's dirty bed sheets.		1	2	3	4	5
Help your loved one take a bath.		1	2	3	4	5
Clean up after your loved one who has lost bowel or bladder control.		1	2	3	4	5
Help your loved one eat a meal.		1	2	3	4	5
Clean up when your loved one has thrown up.		1	2	3	4	5
Turn your loved one in bed.		1	2	3	4	5
Change dressings on your loved one's sores.		1	2	3	4	5

Help your loved one in the bathroom.		1	2	3	4	5
Help your loved one move in and out of bed.		1	2	3	4	5

78. Have your thoughts and feelings about COVID-19 influenced how you feel about caregiving in terms of your willingness to care?

- ❑ Yes, I feel more willing to provide care for my loved one
- ❑ Yes, I feel less willing to provide care for my loved one
- ❑ No

79. The World Health Organisation-Five Well-Being Index (WHO-5)

	Over the last two weeks	All of the time	Most of the time	More than half of the time	Less than half of the time	Some of the time	At no time
**1**	I have felt cheerful and in good spirits	5	4	3	2	1	0
**2**	I have felt calm and relaxed	5	4	3	2	1	0
**3**	I have felt active and vigorous	5	4	3	2	1	0
**4**	I woke up feeling fresh and rested	5	4	3	2	1	0
**5**	My daily life has been filled with things that interest me	5	4	3	2	1	0

80. Caregiver GAINS scale (Pearlin, 1988)

As a result of providing care, to what extent did you:

	A lot	Somewhat	A little	Not at all
Become more sensitive to persons with disabilities?				
Get a better idea of what's important in life?				
Become aware of inner strengths?				
Become closer to God?				

Grow as a person?				
Become closer to family?				
Gain a sense of fulfilling duty?				
Become more self confident?				
Learn to do new things?				
Make new friends?				

81. **Zarit Burden Interview (ZBI-12)** (Bedard et al., 2001)

The following is a list of statements which reflect how people sometimes feel when taking care of their loved one.

Do you feel...

	Never	Rarely	Sometimes	Quite frequently	Nearly always
That because of the time you spend with your loved one that you don't have					
Stressed between caring for your loved one and trying to meet other					
Angry when you are around your loved one?					
That your loved one currently affects your relationship with family members					
Strained when you are around your loved one?					
That your health has suffered because of your involvement with your loved					
That you don't have as much privacy as you would like because of your					

That your social life has suffered because you are caring for your loved					
That you have lost control of your life since your loved one's illness?					
Uncertain about what to do about your loved one?					
You should be doing more for your loved one?					
You could do a better job in caring for your loved one?					

82. EQ-5D-5L

Please select the ONE box that best describes your health TODAY.

MOBILITY

- ❑ I have no problems in walking about
- ❑ I have slight problems in walking about
- ❑ I have moderate problems in walking about
- ❑ I have severe problems in walking about
- ❑ I am unable to walk about

Please select the ONE box that best describes your health TODAY.

SELF-CARE

- ❑ I have no problems washing or dressing myself
- ❑ I have slight problems washing or dressing myself
- ❑ I have moderate problems washing or dressing myself
- ❑ I have severe problems washing or dressing myself
- ❑ I am unable to wash or dress myself

Please select the ONE box that best describes your health TODAY.

USUAL ACTIVITIES (e.g. work, study, housework, family or leisure activities)

- ❑ I have no problems doing my usual activities
- ❑ I have slight problems doing my usual activities

- ❑ I have moderate problems doing my usual activities
- ❑ I have severe problems doing my usual activities
- ❑ I am unable to do my usual activities

Please select the ONE box that best describes your health TODAY.

PAIN / DISCOMFORT

- ❑ I have no pain or discomfort
- ❑ I have slight pain or discomfort
- ❑ I have moderate pain or discomfort
- ❑ I have severe pain or discomfort
- ❑ I have extreme pain or discomfort

Please select the ONE box that best describes your health TODAY.

ANXIETY / DEPRESSION

- ❑ I am not anxious or depressed
- ❑ I am slightly anxious or depressed
- ❑ I am moderately anxious or depressed
- ❑ I am severely anxious or depressed
- ❑ I am extremely anxious or depressed

83. VAS

We would like to know how good or bad your health is TODAY.

This scale is numbered from 0 to 100.

100 means the best health you can imagine. 0 means the worst health you can imagine.

Please indicate on the scale how your health is TODAY.

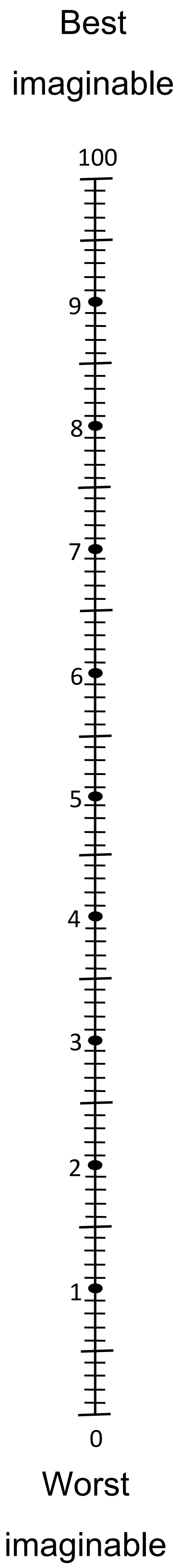

84. **10-item Center for Epidemiologic Studies Depression Scale (CESD-10)** (Andersen et al., 1994)

Below is a list of the ways you might have felt or behaved. Please indicate how often you have felt this way during the past week.

	**Rarely or none of the time (less than 1 day)**	**Some or a little of the time (1-2 days)**	**Occasionally or a moderate amount of time (3-4 days)**	**All of the time (5-7 days)**
I was bothered by things that usually don't bother me				
I had trouble keeping my mind on what I was doing				
I felt depressed				
I felt that everything I did was an effort				
I felt hopeful about the future				
I felt fearful				
My sleep was restless				
I was happy				
I felt lonely				
I could not "get going"				

85. **Dyadic Relationship Scale (DRS)** (Sebern et al. 2007)

This series of questions address some of the difficulties that people face as they care for a relative. We would like to know how helping your loved one has affected your relationship with them during the past week. How much do you agree or disagree with the following statements?

**Because of helping my loved one:**	**Strongly agree**	**Agree**	**Neutral**	**Disagree**	**Strongly disagree**
I felt closer to my loved one than I have in a while.					

I have learned some good things about my loved one.					
I felt angry toward my loved one.					
I felt depressed because of my relationship with my loved one.					
I felt resentful toward my loved one.					
I have had more patience than I have had in the past.					
I have learned some good things about myself.					
I felt that my relationship with my loved one was strained.					
I have learned some nice things about other people in my life.					
Communication between my loved one and me has improved.					
I felt that my loved one made requests over and above what he/she needed.					

86. **Relationship Assessment Scale** - 1 item (Hendrick, 1988)

	**Not satisfied 1**	**2**	**3**	**4**	**Very satisfied 5**
In general, how satisfied are you with your relationship with your loved one?					

Module 1

87. **Do you feel you had a choice in taking on this responsibility of caring for your loved one?**

- ❑ No
- ❑ Yes

88. **Revised Familism Scale (RFS)** (Losada et al., 2020)

The following questions examine family relationships. Please rate on the scale how much you agree or disagree with each statement:

	**Strongly disagree**	**Disagree**	**Neither agree nor disagree**	**Agree**	**Strongly agree**
1 - One should make great sacrifices in order to guarantee a good education for his/her children.					
2 - I would help within my means if a relative told me that they are in financial difficulty.					
3 - One should have the hope of living long enough to see his/her grandchildren grow up.					
4 - A person should share his/her home with uncles, aunts or first cousins if they are in need.					
5 - When someone has problems s/he can count on the help of relatives. (Generally)					
6 - When one has problems, one can count on the help of relatives. (Oneself/In relation to self)					
7 - One can count on help from his/her relatives to solve most problems.					
8 - Much of what a son or daughter does should be done to please the parents.					
9 - The family should consult close relatives (uncles, aunts) concerning its important decisions.					

10 - One should be embarrassed about the bad things done by his/her brothers or sisters.					
11 - Children should live in their parents' house until they get married.					
12 - A person should help his or her elderly parents in times of need, for example, help financially or share a house.					
13 - A person should live near his or her parents and spend time with them on a regular basis.					
14 - A person should always support members of the extender family, for example, aunts, uncles, and in-laws, if they are in need even if it is a big sacrifice.					
15 - A person should respect his or her older brothers and sisters regardless of their differences in views.					
16 - A person should be a good person for the sake of his or her family.					
17 - Children should obey their parents without question even if they believe they are wrong.					
18 - Parents and grandparents should be treated with great respect regardless of their differences in views					
19 - A person should often do activities with his or her immediate and extended families, for example,					

eat meals, play games, go somewhere together, or work on things together.					
20 - The family should control the behavior of children younger than 18.					
21 - A person should cherish time spent with his or her relatives.					

89. The Brief Illness Perception Questionnaire (B-IPQ) / Different format (Broadbent et al. 2006)

For the following questions, please select the number that best corresponds to your views:

**How much does your loved one's illness affect your life?**<br>1 2 3 4 5 6 7 8 9 10<br>no affect at all — severely affects my life
**How long do you think your loved one's illness will continue?**<br>1 2 3 4 5 6 7 8 9 10<br>a very short time — forever
**How much control do you feel you have over your loved one's illness?**<br>1 2 3 4 5 6 7 8 9 10<br>absolutely no control — extreme amount of control
**How much do you think your loved one's treatment can help their illness?**<br>1 2 3 4 5 6 7 8 9 10<br>not at all — extremely helpful
**How much does your loved one experience symptoms from their illness?**<br>1 2 3 4 5 6 7 8 9 10<br>no symptoms at all symptoms — many severe
**How concerned are you about your loved one's illness?**<br>1 2 3 4 5 6 7 8 9 10<br>not at all concerned — extremely concerned

**How well do you feel you understand your loved one's illness?**<br>1 2 3 4 5 6 7 8 9 10<br>don't understand understand<br>at all very clearly
**How much does your loved one's illness affect you emotionally? (e.g. does it make you angry, scared, upset or depressed?**<br>1 2 3 4 5 6 7 8 9 10<br>not at all extremely<br>affected affected<br>emotionally emotionally
**Please list in rank-order the three most important factors that you believe caused <u>your loved one's illness</u>. *The most important causes for me:***<br><br>**1.** _<br>**2.** _<br>**3.** __

90. Meaning in Life Questionnaire (MLQ) (Steger et al., 2006)

Please take a moment to think about what makes your life and existence feel important and significant to you. Please respond to the following statements as truthfully and accurately as you can, and also please remember that these are very subjective questions and that there are no right or wrong answers. Please answer according to the scale below:

	**Absolutely untrue**	**Mostly Untrue**	**Somewhat Untrue**	**Can't Say True or False**	**Somewhat True**	**Mostly True**	**Absolutely True**
I understand my life's meaning.<br>I am looking for something that makes my life feel meaningful.							
I am always looking to find my life's purpose.							

My life has a clear sense of purpose.							
I have a good sense of what makes my life meaningful.							
I have discovered a satisfying life purpose.							
I am always searching for something that makes my life feel significant.							
I am seeking a purpose or mission for my life.							
My life has no clear purpose.							
I am searching for meaning in my life.							

91. Portrait Values Questionnaire (PVQ-21) (Schwartz et al., 2001)

Here we briefly describe some people. Please read each description and think about how much each person is or is not like you.

How much is this person like you?

	very much like me	like me	somewhat like me	a little like me	not like me	not like me at all
It is important to him/her to be rich. He/she wants to have a lot of money and expensive things.						
He/she thinks it is important that every person in the world should be treated equally. He/she believes everyone should have equal opportunities in life.						
It's very important to him/her to show his/her abilities. He/she wants people to admire what he/she does.						
It is important to him/her to listen to people who are different from him/her. Even when he/she disagrees with them, he/she still wants to understand them.						
It's very important to him/her to help the people around him/her. He/she wants to care for their well-being.						
Being very successful is important to him/her. He/she hopes people will recognize his/her achievements.						
It is important to him/her to get respect from others. He/She wants people to do what he/she says.						
It is important to him/her to be loyal to his/her friends. He/she wants to devote himself/herself to people close to him/her.						
He/she strongly believes that people should care for nature. Looking after the environment is important to him/her.						

Module 2

Here are some questions about your loved one's living arrangements and the geographic distance between you and them. Please read each of the questions carefully and choose the answer that best applies to your loved one's situation.

Skip to question 100 if you answered "yes" to question 36

92. **Over the past month, how often did you visit your loved one?**

- ❑ Daily
- ❑ More than once a week
- ❑ Once a week
- ❑ Less than once a week

93. **Over the past 3 months, how often did you feel unable to continue caring for your loved one because you don't live together?**

- ❑ Never
- ❑ Rarely
- ❑ Sometimes
- ❑ Often
- ❑ Always

94. **Travel time to visit your loved one is:**

- ❑ Greater than the time actually spent visiting
- ❑ Equal to the time spent visiting
- ❑ Shorter than the time spent visiting

95. **To what extent is the geographic distance between you and your loved one, a barrier to your investment in their care?**

- ❑ Not at all
- ❑ A little
- ❑ Somewhat
- ❑ A lot

96. **Do you feel that the travel time to your loved one, negatively impacts your personal daily activities ?**

For example, housework, shopping, social outings, religious activities, etc.

- ❑ Not at all
- ❑ A little
- ❑ Somewhat
- ❑ A lot

97. **Since the onset of care dependency, have you ever considered moving to shorten the geographic distance between you and your loved one?**

- ❑ No
- ❑ Yes

98. Do you feel that the geographic distance between you and your loved one, negatively impacts your relationship with them?

- ❑ Not at all
- ❑ A little
- ❑ Somewhat
- ❑ A lot

99. Over the past 3 months, how often did you feel like leaving the care of your loved one to someone else, due to the travel time it takes to visit them?

- ❑ Never
- ❑ Rarely
- ❑ Sometimes
- ❑ Often
- ❑ Always

We would now like to ask you some questions relating to the mode of transportation you are using and the help that your loved one might receive from you, regarding their transportation needs.

100. What is your primary mode of transportation during a typical week?

Public transport includes: bus, coach, tram and metro

- ❑ Walking
- ❑ Bicycle
- ❑ Car
- ❑ Motorcycle
- ❑ Train
- ❑ Public transport

101. Is your loved one currently able to get to the services they are using or activities by themselves?

For example, drive or use public transportation to medical services, shopping or errands, social activities, family or personal business, religious activities, etc.

- ❑ No, they need assistance.
- ❑ Yes (Skip to question 103)

102. Please indicate the reason(s) why your loved one needs assistance to get to these services or activities:

Please select all that apply.

- ❑ Diagnosed Health condition/s as indicated earlier
- ❑ Hearing impairment
- ❑ Vision impairment
- ❑ Reduced mobility
- ❑ Prescription drug side effects
- ❑ They do not have a driver's license
- ❑ Old age
- ❑ If Other, please specify

103. *Skip to question 104 if you answered "no" to question 101*

What is your loved one's primary mode of transportation during a typical week?

Public transport includes: bus, coach, tram and metro

- ❑ Walking
- ❑ Bicycle
- ❑ Car
- ❑ Motorcycle
- ❑ Train
- ❑ Public transport

104. Over the past 3 months, how much did you assist your loved one to get to the following services they are using or activities?

For example, assisting by driving them to these services/activities or helping them with using public transportation.

Not Applicable option is provided in case some of these services/activities are irrelevant to your loved one's circumstances or you don't personally assist them to get to these services/activities

	**For all of their trips**	**For about 75% of their trips**	**For about 75% of their trips**	**For about 75% of their trips**	**For about 75% of their trips**	**For about 75% of their trips**
Medical services						
Shopping or errands						

Social and recreational activities						
Family or personal business						
Religious activities						

105. How long does it take you to get your loved one to the following services/activities?

Not Applicable option is provided in case some of these services/activities are irrelevant to your loved one's circumstances or you don't personally assist them to get to these services/activities

	**Less than 15 minutes**	**Less than 15 minutes**	**Less than 15 minutes**	**Less than 15 minutes**	**Less than 15 minutes**	**Less than 15 minutes**
Medical services						
Shopping or errands						
Social and recreational activities						
Family or personal business						
Religious activities						

Skip to question 108 if you answered "Yes" to question 36

106. Is there available parking near your loved one's residence?

- ❑ No
- ❑ Yes

107. To what extent, is the availability of parking near your loved one's residence, a barrier to your investment in their care?

- ❑ Not at all

- ❑ A little
- ❑ Somewhat
- ❑ A lot

108. Over the past 3 months, how often did you feel like leaving the care of your loved one to someone else, due to their transportation needs?

- ❑ Never
- ❑ Rarely
- ❑ Sometimes
- ❑ Often
- ❑ Always

109. Do you feel that the transportation needs of your loved one, negatively impact your personal daily activities?

For example, housework, shopping, social outings, religious activities etc.

- ❑ Not at all
- ❑ A little
- ❑ Somewhat
- ❑ A lot

We would now like to ask you some questions relating to the support services you, the caregiver, received in the past 6 months.

Answer questions 110 and 111 if you selected that you have received coordination services on question 69

110. You indicated that you have received coordination services providing information about the help, support and benefits that is available and how to access it. How far is your home from these services?

- ❑ Less than 15 minutes
- ❑ 15 to 30 minutes
- ❑ 30 to 60 minutes
- ❑ 60 to 90 minutes
- ❑ 90 to 120 minutes
- ❑ More than 120 minutes

111. Over the past 6 months, how often did you feel discouraged from using these coordination services due to the travel time it needs to get there?

- ❑ Never
- ❑ Rarely
- ❑ Sometimes

- ❑ Often
- ❑ Always

Answer questions 112 and 113 if you selected that you have received information services on question 69

112. You indicated that you have received services providing information about the disease that your loved one has. How far is your home from these services?

- ❑ Less than 15 minutes
- ❑ 15 to 30 minutes
- ❑ 30 to 60 minutes
- ❑ 60 to 90 minutes
- ❑ 90 to 120 minutes
- ❑ More than 120 minutes

113. Over the past 6 months, how often did you feel discouraged from using these services that provide information about the disease that your loved one has, due to the travel time it needs to get there?

- ❑ Never
- ❑ Rarely
- ❑ Sometimes
- ❑ Often
- ❑ Always

Answer questions 114 and 115 if you selected that you have received counselling services on question 69

114. You indicated that you have received counselling services. How far is your home from these services?

- ❑ Less than 15 minutes
- ❑ 15 to 30 minutes
- ❑ 30 to 60 minutes
- ❑ 60 to 90 minutes
- ❑ 90 to 120 minutes
- ❑ More than 120 minutes

115. Over the past 6 months, how often did you feel discouraged from using these counselling services due to the travel time it needs to get there?

- ❑ Never
- ❑ Rarely
- ❑ Sometimes
- ❑ Often
- ❑ Always

Answer questions 116 and 117 if you selected that you have received self-help or support group services on question 69

116. You indicated that you have received self-help or support group services. How far is your home from these services?

- ❑ Less than 15 minutes
- ❑ 15 to 30 minutes
- ❑ 30 to 60 minutes
- ❑ 60 to 90 minutes
- ❑ 90 to 120 minutes
- ❑ More than 120 minutes

117. Over the past 6 months, how often did you feel discouraged from using these self-help or support services due to the travel time it needs to get there?

- ❑ Never
- ❑ Rarely
- ❑ Sometimes
- ❑ Often
- ❑ Always

Answer questions 118 and 119 if you selected that you have received training services on question 69

118. You indicated that you have received training to help you develop the skills you need to provide care and cope with the burden of care. How far is your home from these services?

- ❑ Less than 15 minutes
- ❑ 15 to 30 minutes
- ❑ 30 to 60 minutes
- ❑ 60 to 90 minutes
- ❑ 90 to 120 minutes
- ❑ More than 120 minutes

119. Over the past 6 months, how often did you feel discouraged from using these training services due to the travel time it needs to get there?

- ❑ Never
- ❑ Rarely
- ❑ Sometimes
- ❑ Often
- ❑ Always

120. In your opinion, which service(s) would most help you as a caregiver?

Please select all that apply.

- ❑ Services providing information about the disease that your loved one has
- ❑ Coordination services providing information and advice about the type of help, support and benefits that is available to carers and how to acce

- ❑ Training services to help carers develop the skills they need to care and cope with the burden of care
- ❑ Counselling services (i.e. supportive and social counselling, etc.)
- ❑ Self-help or support group services
- ❑ If Other, please specify

121. How far would you be willing to travel for this service(s)?

- ❑ Less than 15 minutes
- ❑ 15 to 30 minutes
- ❑ 30 to 60 minutes
- ❑ 60 to 90 minutes
- ❑ 90 to 120 minutes
- ❑ More than 120 minutes
- ❑ I would prefer to receive these services online

122. Over the past 3 months, how often did you feel that you lack information about your loved one's condition?

For example, information about the course of their condition, their medical care, their medical exams results, etc.

- ❑ Never
- ❑ Rarely
- ❑ Sometimes
- ❑ Often
- ❑ Always

123. The Relationship Structures Questionnaire of the Experiences in Close Relationships - Revised (ECR-RS)

(Fraley et al., 2011)

Please read each of the following statements carefully and rate the extent to which you believe each statement best describes your feelings about your loved one.

It helps to turn to my loved one in times of need.

strongly disagree 1 2 3 4 5 6 7 strongly agree

I usually discuss my problems and concerns with my loved one.

strongly disagree 1 2 3 4 5 6 7 strongly agree

I talk things over with my loved one.

strongly disagree 1 2 3 4 5 6 7 strongly agree

I find it easy to depend on my loved one.

strongly disagree 1 2 3 4 5 6 7 strongly agree

I don't feel comfortable opening up to my loved one.

strongly disagree 1 2 3 4 5 6 7 strongly agree

I prefer not to show my loved one how I feel deep down.

strongly disagree 1 2 3 4 5 6 7 strongly agree

I often worry that my loved one doesn't really care for me.

strongly disagree 1 2 3 4 5 6 7 strongly agree

I'm afraid that my loved one may abandon me.

strongly disagree 1 2 3 4 5 6 7 strongly agree

I worry that my loved one won't care about me as much as I care about them.

strongly disagree 1 2 3 4 5 6 7 strongly agree

---

124. Big-Five Inventory Extra Short Form (BFI-2-XS) (Soto & John, 2017)

Here are a number of characteristics that may or may not apply to you. For example, do you agree that you are someone who likes to spend time with others? Please read each of the statements carefully and rate the extent to which you agree or disagree.

I am someone who...

	**Disagree strongly**	**Disagree a little**	**Neutral; no opinion**	**Agree a little**	**Agree strongly**
Tends to be quiet.					
Is compassionate, has a soft heart.					
Tends to be disorganized.					
Worries a lot.					
Is fascinated by art, music, or literature.					
Is dominant, acts as a leader.					
Is sometimes rude to others.					

Has difficulty getting started on tasks.					
Tends to feel depressed, blue.					
Has little interest in abstract ideas.					
Is full of energy.					
Assumes the best about people.					
Is reliable, can always be counted on.					
Is emotionally stable, not easily upset.					
Is original, comes up with new ideas.					

---

125. The Pearlin Mastery Scale (Pearlin & Schooler, 1978)

On a scale of 1 to 4 with 1 meaning "Strongly Disagree" and 4 meaning "Strongly Agree", how strongly do you agree or disagree with these statements about yourself?

	**Strong Disagree**	**Disagree**	**Agree**	**Strongly Agree**
There is really no way I can solve some of the problems I have.				
Sometimes I feel that I'm being pushed around in life.				
I have little control over the things that happen to me.				
I can do just about anything I really set my mind to.				
I often feel helpless in dealing with the problems of life.				

What happens to me in the future mostly depends on me.				
There is little I can do to change many of the important things in my life.				

---

126. Toronto Empathy Questionnaire (TEQ) (Spreng et al., 2009)

Below is a list of statements. Please read each of the statements carefully and rate how frequently you feel or act in the manner described. There are no right or wrong answers or trick questions. Please answer each question as honestly as you can.

	**Never**	**Rarely**	**Sometimes**	**Often**	**Always**
When someone else is feeling excited, I tend to get excited too.					
Other people's misfortunes do not disturb me a great deal.					
It upsets me to see someone being treated disrespectfully.					
I remain unaffected when someone close to me is happy.					
I enjoy making other people feel better.					
I have tender, concerned feelings for people less					

fortunate than me.					
When a friend starts to talk about his\her problems, I try to steer the conversation towards something else.					
I can tell when others are sad even when they do not say anything.					
I find that I am “in tune” with other people’s moods.					
I do not feel sympathy for people who cause their own serious illnesses.					
I become irritated when someone cries.					
I am not really interested in how other people feel.					
I get a strong urge to help when I see someone who is upset.					
When I see someone being treated unfairly, I do not feel very much pity for them.					

I find it silly for people to cry out of happiness.					
When I see someone being taken advantage of, I feel kind of protective towards him/her.					

Module 3

127. Perception of Collaboration Questionnaire (PCQ) (Berg et al., 2008)

Here are some questions about perceptions of collaboration between you and your loved one. Please indicate the extent to which you agree or disagree with that statement

	Strongly disagree	Disagree	Neutral	Agree	Strongly agree
It is necessary for my loved one and I to work together, as it is harder for my loved one to solve problems and make decisions by him/herself					
My loved one makes better decisions when we work together					
My loved one and I always work together to deal with care-related decisions					

Nearly every day my loved one and I work together to make care-related decisions					
It is rare for my loved one and I to share care-related tasks and make decisions together					
My loved one enjoys the support and encouragement he/she receives when we work together					
Working together with my loved one is useful as I make up for things that my loved one doesn't do well					
Solving everyday problems and making decisions together with my loved one brings us closer together					
My loved one dislikes my assistance as it makes him/her feel incompetent					

128. Dyadic Coping Inventory (DCI) - stress communication subscale (Bodenmann, 2000)

Please respond to any item by marking the appropriate case, which is fitting to your personal situation.

This section is about how you communicate your stress with your loved one:

	Very rarely	Rarely	Sometimes	Often	Very often
I let my loved one know that I appreciate his/her practical support, advice, or help					
I ask my loved one to do things for me when I have too much to do					
I show my loved one through my behaviour when I am not doing well or when I have problems					
I tell my loved one openly how I feel and that I would appreciate his/her support					

This section is about how your loved one communicates when he/she is feeling stressed:

	Very rarely	Rarely	Sometimes	Often	Very often
My loved one lets me know that he/she appreciates my practical support, advice, or help					
My loved one asks me to do things for					

him/her when he/she has too much to do					
My loved one shows me through his/her behaviour that he/she is not doing well or when he/she has problems					
My loved one tells me openly how he/she feels and that he/she would appreciate my support					

129. Mutuality scale (MS) (Archbold et al., 1990)

Now we would like you to let us know how you and your loved one feel about each other at the current time.

	Not at all	A little	Some	Quite a bit	A great deal
To what extent do the two of you see eye to eye (agree on things)?					
How close do you feel to your loved one?					
How much do you enjoy sharing past experiences with your loved one?					

How much does your loved one express feelings of appreciation for you and the things you do?					
How attached are you to your loved one?					
How much does your loved one help you?					
How much do you like to sit and talk with your loved one?					
How much love do you feel for your loved one?					
To what extent do the two of you share the same values?					
When you really need it, how much does your loved one comfort you?					
How much do the two of you laugh together?					
How much do you confide in your loved one?					

How much emotional support does your loved one give you?					
To what extent do you enjoy the time the two of you spend together?					
How often does your loved one express feelings of warmth toward you?					

130. The perceived partner responsiveness scale (PPRS) (Reis et al., 2018)

Please answer the following questions about your loved one.

My loved one usually:

	Not at all true		Somewhat true		Moderately true		Very true		Completely true
really listens to me									
is responsive to my needs									
sees the "real" me									
"gets the facts right" about me									
understands me									
is on the “same wavelength” with me									
knows me well									

esteems me, shortcomings and all									
values and respects the whole package that is the "real" me									
expresses liking and encouragement for me									
seems interested in what I am thinking and feeling									
values my abilities and opinions									

131. **Social Support List (SSL) (**Kempen & Van Eijk, 1995; Van Sonderen, 1993)

The next questions concern the help and support you provide to your loved one. Please keep your loved one in mind.

How often do you:

	Seldom or never	Now and then	Quite often	Very often
Provide attention to your loved one				
Provide support to your loved one				
Show that you appreciate your loved one				
Provide instrumental support to your loved one				

Keep your loved one company				
Provide information and advice to your loved one				
React in a distant manner to your loved one				
Do not keep your appointments with your loved one				
Make disapproving remarks to your loved one				
Blame or holding things against your loved one				
Treat your loved one unfairly				
Make unreasonable demands to your loved one				
Interfere too much with your loved one				

The next questions concern the help and support you receive from your loved one. Please keep your loved one in mind.

How often does your loved one:

	Seldom or never	Now and then	Quite often	Very often
Provide attention to you				
Provide support to you				
Show that he/she appreciates you				

Provide instrumental support to you				
Keep you company				
Provide information and advice to you				
React in a distant manner to you				
Do not keep his/her appointments with you				
Make disapproving remarks to you				
Blame or holding things against you				
Treat you unfairly				
Make unreasonable demands to you				
Interfere too much with you your loved one				

Module 4

132. Do you receive financial compensation from your loved one or another family member for the care that you provide?

- ❑ No
- ❑ Yes: _____ per month.

Skip to question 135 if you answered "no" to "Your work/employment situation (i.e. stopped or started working, change in number of working hours, change in income from employment, etc...)" in question 9 and "yes" to question 20 or 23

We will now ask you questions regarding your current work status.

133. **Did you reduce your working hours due to your loved one's disease/condition (e.g. to care for your loved one)?**

- ❑ No
- ❑ Yes

134. **Did you stop working because of caregiving?**

- ❑ No (skip to question 136)
- ❑ Yes. How many hours per week did you used to work? _______hours

135. **How many hours per week did you work before reducing your working hours?**

________ hours per week.

136. *Skip to question 139 if you answered "no" to question 20 or 23*

Please answer the following questions in relation to the last week OR on a typical week if last week was a holiday

During the last week, how many hours did you miss from work due to your loved one's disease/condition?

Include hours missed when you came in late or left work early because you e.g. accompanied your loved one to doctor appointments, visited hospitals or clinics, or helped them with dressing, grooming, eating, or take medications.

- ❑ I did not miss any hours of work during the last week due to my loved one condition.
- ❑ I missed ____hours of work last week due to my loved one condition (skip to question 138)

137. **Was any of this time missed from work unpaid?**

- ❑ No, all the time I missed from work was paid.
- ❑ Yes, _____ hour(s) were unpaid.

138. **During the last week, how much did your loved one's disease/condition affect your productivity while you were working?**

If you were able to work as usual, choose a low number. If you were not able to work as usual (e.g. accomplished less than usual, could not concentrate or perform certain tasks as carefully as usual), choose a high number.

**Could work as usual**										**Could not work at all**
**0**	**1**	**2**	**3**	**4**	**5**	**6**	**7**	**8**	**9**	**10**
☐	☐	☐	☐	☐	☐	☐	☐	☐	☐	☐

139. Skip to question 147 if you answered "no" to question 57

We would now like to ask you some questions relating to the use of home care services and the decision to hire paid care workers

Please indicate the home care services that your loved one has received in the last month.

Please select all that apply.

- ❑ Help with personal care (e.g. dressing/undressing, washing/showering/bathing, hair care, shaving and grooming, and going to the toilet)
- ❑ Nursing care (e.g. administering medications and injections, monitoring vital signs, dressing wounds, performing medical therapies, nutrition via tubes, prevention and management of bed sores and any medical-related types of tasks)
- ❑ Help with household activities and tasks (e.g. preparing food, cleaning, washing, ironing, sewing, shopping, and gardening)
- ❑ Practical support (e.g. eating and drinking, moving inside or outside the house (including assistance with walking or using a wheelchair), visiting family or friends, accompany to healthcare visits (e.g. doctor appointments), filling prescriptions at the pharmacy, help taking medications, and taking care of financial matters (e.g. paying the bills or managing healthcare insurance)
- ❑ Sitting services (provide company and support to the dependent when a caregiver is temporarily absent)
- ❑ Home delivered meals/ Meals-on-wheels (ready-made meals provided by a municipality or a private provider)
- ❑ Telecare (e.g alarm buttons, help lines, bed monitors, video communication systems, fall and movement detectors, smartphone apps or any other technologies that assist your loved one to continue living at home)
- ❑ Other (specify below)
- ❑ None (skip to question 141)

140. In a typical week, how many hours of the following home care service(s) does your loved one receive from one or more paid care workers?

Please note that the home care services have been separated into services provided by a national care worker, and those provided by a migrant care workers.

To avoid overlap in the number of hours provided, if you believe that a task fits into more than one category, please select the category that you believe best fits the task.

Only fill in the rows that correspond to the selections made in question 140

Home care service	In a typical week, how many hours of this service has been provided by a **national care worker**?	*[Only complete this column if the person you provide care for receives care from a migrant care worker]* In a typical week, how many hours of this service has been provided by a **migrant care worker**?
1. Help with **personal care** (e.g. assistance with dressing/undressing, washing, combing, shaving, going to the toilet, moving around in the house, eating, drinking and administering medication and any other non-medical types of tasks).		
2. **Nursing care**. That would include administering medications and injections, monitoring vital signs, dressing wounds, performing medical therapies, nutrition via tubes, prevention and management of bed sores and any medical-related types of tasks.		

3. Help with **household activities and tasks** (e.g. assistance with preparing food and drinks, cleaning the house, washing, ironing and sewing clothing, shopping for groceries or odd jobs in the house or the garden and any other household chores your loved one needs support with).		
4. **Practical support** (e.g. moving around outside the house, going on outings and visiting family or friends, contacts with healthcare (accompanying your loved one for example to the general practitioner, the hospital, therapy), arranging assistance, devices and/or home modifications and organising financial and administrative matters.		
5. Sitting services (provide company and support to the dependent when a caregiver is temporarily absent)		
6. Home delivered meals/ Meals-on-wheels (i.e. ready-made meals provided by a municipality or a private provider)		

Telecare (i.e alarm buttons, help lines, bed monitors, video communication systems, fall and movement detectors, smartphone apps or any other technologies that assist your loved one to continue living at home).	[redacted]

141. Why did you decide to hire a private care worker?

Please select all that apply.

- ❑ It gives me more time to myself/for other tasks
- ❑ They can provide expertise/support that I can't
- ❑ I am not able to provide care
- ❑ To guarantee constant care
- ❑ To keep my same life style
- ❑ To safeguard my own family
- ❑ I live too far away/ too much travel
- ❑ To provide the necessary amount of care
- ❑ There are a lack of other alternatives
- ❑ So I am able to keep on working
- ❑ It is cheaper than other alternatives
- ❑ I am not willing to provide care
- ❑ My loved one was willing to have a cohabiting care worker
- ❑ If Other, please specify

Skip to question 143 if you answered "yes" to question 59 or question 64

142. Why was it decided not to hire a migrant worker?

Please select all that apply.

- ❑ I would not trust a migrant worker to take care of my loved one
- ❑ Not able to afford this service
- ❑ I prefer that the care worker is a national citizen
- ❑ My loved one refused this solution
- ❑ Do not know
- ❑ If Other, please specify

Skip to question 146 if you answered "no" or "do not know" to question 59 or question 64

143. Why was it decided to hire a migrant worker?

Please select all that apply.

- ❑ Economically convenient
- ❑ It is useful for both myself and my loved one
- ❑ Migrant workers provide care tasks that local staff are not able to provide (for example late night shifts/weekends).
- ❑ It is common practice
- ❑ To avoid having my loved one live in a residential facility
- ❑ Do not know
- ❑ If Other, please specify

144. Were you involved in the decision to hire a migrant worker?

- ❑ No
- ❑ Yes

145. How did your loved one access services by a migrant care worker?[3]

Please select all that apply

- ❑ Through recommendations from family or friends
- ❑ Through a care or recruitment agency
- ❑ Through a church or religious organization
- ❑ Through a government programme
- ❑ Through a non-profit organization
- ❑ Through social media
- ❑ Other, please specify:

We would now like to ask you some questions relating to the support service that you, the caregiver, received in the past 6 months

Only answer question 146 if you selected "Information about the disease that the loved one has" on question 69

146. You indicated that you have received services providing information about the disease that your loved one has.

Do you pay for this service when you utilise it?

- ❑ No

[3] Question not asked to Swedish participants as the question was added after ethics approval in Sweden

- ❑ Yes. How much do you pay for this service when you utilise it?______.

Only answer question 147 if you selected "Coordination services providing information and advice about the type of help, support and benefits that is available to carers and how to access it (e.g. information centers, case (or care) managers, advice or guidance from a physician or social worker" on question 69

147. You indicated that you have received coordination services providing information about the help, support and benefits that is available and how to access it. Do you pay for this service when you utilise it?

- ❑ No
- ❑ Yes. How much do you pay for this service when you utilise it?______.

Only answer question 148 if you selected "Training to help carers develop the skills they need to care and cope with the burden of care" on question 69

148. You indicated that you have received training to help you develop the skills you need to care. Do you pay for this service when you utilise it?

- ❑ No
- ❑ Yes. How much do you pay for this service when you utilise it?______.

*Only answer question 149 if you selected "*Counselling (i.e. supportive and social counselling, etc.)*" on question 69*

149. You indicated that you have received counselling services. Do you pay for this service when you utilise it?

- ❑ No
- ❑ Yes. How much do you pay for this service when you utilise it?______.

150. In this section we would like to find out about any financial costs that you may have had as a result of caring for your loved one

Please only include expenses you pay for with your own money or with household money and do not count expenses that are covered or reimbursed by a health or social insurance

- **travel to visit your loved one or to take them to appointments at hospital, hospice, GP surgery or elsewhere?**

e.g. taxis, buses, trains, planes, parking, fuel if you travel in your own car, accommodation (to be close to the hospital, hospice or elsewhere), or any other incurred travel expenses.

- ❑ No
- ❑ Yes. How much did you pay in the last month? _______

- **for childcare or care for any other dependents?**

e.g. paying someone to look after children or any other dependents while you were caring for your loved one.

- ❑ No
- ❑ Yes. How much did you pay in the last month? _______

.

- **medical care for your loved one?**

e.g. health or long-term care insurance premiums, medical consultations, physio, private ambulance, hospital admissions, prescription medicines, over the counter medications or supplements (e.g. paracetamol, vitamins, etc.), complementary or alternative medicines/therapies (i.e chiropractic and massage therapy).

- ❑ No
- ❑ Yes. How much did you pay in the last month? _______

- **your loved one's food, meals, household goods, incidentals or clothing?**

- ❑ No
- ❑ Yes. How much did you pay in the last month? _______.

- **your loved one's housing payments, rent, utilities or home maintenance and repairs?**

e.g. electricity, water, heating, phone, internet and yard care.

- ❑ No
- ❑ Yes. How much did you pay in the last month? _______

- **home care or in-home respite care services?**

*In-home respite care is temporary or short-term care of an individual that is provided at their home by someone other than the person's normal caregiver.

- ❑ No
- ❑ Yes. How much did you pay in the last month? _______

- **out-of-home respite services for your loved one**?

e.g. day-care services and temporary stay at a residential or nursing home.

- ❑ No
- ❑ Yes. How much did you pay in the last month? _______

151. In the last month, have you had out-of-pocket expenses for any of the following aids, appliances and modifications due to your loved one's condition?

- **walking aids (e.g. stick/crutch, walker, cane, wheelchair, scooter)?**

  - ❑ No
  - ❑ Yes. How much did you pay in the last month? _______

- **home living aids (e.g. lifting/reclining armchair, stair lift, bath lift, shower seat, raisers for bed)?**

  - ❑ No
  - ❑ Yes. How much did you pay in the last month? _______

- **home or car modifications (e.g. ramps, rails, home extensions, wheel chair lift, boot hoist)?**

  - ❑ No
  - ❑ Yes. How much did you pay in the last month? _______

- **personal alarms or monitoring systems (e.g. webcams, intercom and tracking systems)?**

  - ❑ No
  - ❑ Yes. How much did you pay in the last month? _______

- **other aids or appliances?**

For example, incontinence aids (pads, pants, waterproof sheets/chair covers), respiratory aids (e.g. home oxygen, NIV machine), posture aids (e.g. back sup

- ❑ No
- ❑ Yes. How much did you pay in the last month? _______

152. In the last month, have you incurred any costs relating to health problems of your own that have occurred as a result of your caring role e.g.

- ❑ No (skip to end of questionnaire)
- ❑ Yes. How much did you pay in the last month? _______

153. Please specify the type and amount of the cost(s) relating to health problem(s) of your own that have occurred as a result of your caring role

Nature of cost e.g. physio for back injury	Cost in in the last month

1	
2	
3	
4	
5	
6	
7	
8	

Thank you for completing our survey.

We very much appreciate the information that you have provided and your time taken in completing this survey. You can find the latest updates on our project at: https://www.entwine-icohort.eu/

If you have any further questions on the survey, please send us an email at: entwinestudy@bangor.ac.uk
