# Supplementary material for: A Multinational Longitudinal Study Incorporating Intensive Methods to Examine Caregiver Experiences in the Context of Chronic Health Conditions: Protocol of the ENTWINE-iCohort
Source: Int J Environ Res Public Health. 2022 Jan 12;19(2):821. doi: 10.3390/ijerph19020821 (PMC8775526; doi:10.3390/ijerph19020821)
Supplement: Supplementary file 1 [file ijerph-19-00821-s001.zip › Supplementary File S7. ENTWINE-iCOHORT Care Recipient Follow up Survey.pdf]

ENTWINE

INFORMAL CARE

The European Training Network on Informal Care

ENTWINE-iCOHORT CARE RECIPIENT FOLLOW-UP SURVEY

This study is being carried out by Bangor University (UK), University Medical Center of Groningen (The Netherlands), National Institute for Health and Science on Ageing (Italy), Bar-Ilan University (Israel) and Uppsala University (Sweden).

About six months ago you filled in a survey relating to your experiences in receiving care. You may notice that many of the questions in this survey are the same as the questions in the initial questionnaire. We ask the same questions, as by monitoring any changes in your experiences and the experiences of your loved one, we can create a more nuanced understanding of the long term caring needs and challenges of informal caregivers. This will be crucial in developing new interventions and policies to support informal caregivers.

Instructions for this questionnaire:

- If you read **'your loved one'**, then the question refers to your caregiver (the same person you referred to in the first questionnaire six months ago).
- Read each question through completely before answering.
- "Other" option is provided in case you believe that the response options do not describe your situation adequately. In this case, please specify your answer by filling in the blank space next to it.
- If you need to take breaks while completing the survey, you can close the survey window at any point and your responses will be automatically saved. When you are ready to continue, simply click on the survey link you received by email and you will then be taken back to the last question you had answered before you exited during your previous session.
- We recommend that you fill in this questionnaire using a laptop or tablet.

We thank you in advance for your valuable time taken to fill in this questionnaire!

1. **Please select from the list below your current country of residence.**
   - ❑ UK
   - ❑ the Netherlands
   - ❑ Greece
   - ❑ Italy
   - ❑ Ireland
   - ❑ Poland
   - ❑ Sweden
   - ❑ Germany
   - ❑ Israel
   - ❑ Other (specify): ______________

2. **Are you still receiving care from the same person you referred to in the first questionnaire approximately six months ago?**
   - ❑ No
   - ❑ Yes (skip to question 9)

3. **Why are you no longer receiving care from your loved one?**

- ❑ My loved one died
- ❑ I am being cared for by another caregiver
- ❑ I no longer need care
- ❑ I have entered formal residential care
- ❑ Other (specify): ________________
- ❑ Prefer not to say

4. **In the future, how willing would you be to receive care from an informal caregiver?**

- ❑ Completely unwilling
- ❑ Somewhat unwilling
- ❑ Not sure
- ❑ Somewhat willing
- ❑ Completely willing

5. **The World Health Organisation-Five Well-Being Index (WHO-5)**

	Over the last two weeks	All of the time	Most of the time	More than half of the time	Less than half of the time	Some of the time	At no time
**1**	I have felt cheerful and in good spirits	5	4	3	2	1	0
**2**	I have felt calm and relaxed	5	4	3	2	1	0
**3**	I have felt active and vigorous	5	4	3	2	1	0
**4**	I woke up feeling fresh and rested	5	4	3	2	1	0
**5**	My daily life has been filled with things that interest me	5	4	3	2	1	0

6. **Meaning in Life Questionnaire (MLQ)** (Steger et al., 2006)

Please take a moment to think about what makes your life and existence feel important and significant to you. Please respond to the following statements as truthfully and accurately as you can, and also please remember that these are very subjective questions and that there are no right or wrong answers. Please answer according to the scale below:

	**Absolutely untrue**	**Mostly Untru**	**Somewhat Untrue**	**Can't Say True**	**Somewhat True**	**Mostly True**	**Absolutely True**

		**e**		**or False**			
I understand my life's meaning. I am looking for something that makes my life feel meaningful.							
I am always looking to find my life's purpose.							
My life has a clear sense of purpose.							
I have a good sense of what makes my life meaningful.							
I have discovered a satisfying life purpose.							
I am always searching for something that makes my life feel significant.							
I am seeking a purpose or mission for my life.							

My life has no clear purpose.							
I am searching for meaning in my life.							

7. **10-item Center for Epidemiologic Studies Depression Scale (CESD-10)** (Andersen et al., 1994)

Below is a list of the ways you might have felt or behaved. Please indicate how often you have felt this way during the past week.

	**Rarely or none of the time (less than 1 day)**	**Some or a little of the time (1-2 days)**	**Occasionally or a moderate amount of time (3-4 days)**	**All of the time (5-7 days)**
I was bothered by things that usually don't bother me				
I had trouble keeping my mind on what I was doing				
I felt depressed				
I felt that everything I did was an effort				
I felt hopeful about the future				
I felt fearful				
My sleep was restless				
I was happy				
I felt lonely				
I could not "get going"				

Please answer question number 8 only if you replied "my loved one died" to question number 3.

8. **To help you deal with feelings of loss, grief, loneliness or missing your loved one, which of the following actions have you taken?**
   Please select all that apply

- ❑ Gone to a self-help group or support group for people who have lost a loved one
- ❑ Spoken to a religious leader
- ❑ Talked to your family doctor
- ❑ Gone to a mental health professional for help—someone like a social worker or psychologist
- ❑ Other (specify): _____________

- ❑ None of the above

9. **Has any of the following changed since you completed the last survey approximately six months ago?**

Please answer “yes” or “no” to each item below.	**No**	**Yes**
Your address		
Your marital/relationship status		
The care benefits you receive, if any. (e.g., cash benefits, tax benefits, or service vouchers)		
Your work/employment situation (i.e. stopped or started working, change in number of working hours, change in income from employment, etc...)		

10. **Since the last assessment, would you say your health condition has:**

- ❑ stayed the same
- ❑ significantly worsened
- ❑ significantly improved

11. **What is your gender?**
- ❑ Female
- ❑ Male
- ❑ Non-binary / third gender
- ❑ Prefer to self describe:
- ❑ Prefer not to say

12. **Which city/town do you live in?**

____

13. **Is your home located in?**
- ❑ central city/area
- ❑ peripheral area in the city
- ❑ rural area

14. **Please indicate the highest level of education you have achieved**
- ❑ Primary
- ❑ Secondary
- ❑ Post-secondary vocational education (e.g., further education college, apprenticeships etc.)

- ❑ Post secondary academic education (e.g., university)
- ❑ Not listed or other

15. What is your marital status?
- ❑ Single (never married) (skip to question 17)
- ❑ Married
- ❑ In a civil union/domestic partnership
- ❑ Divorced (skip to question 17)
- ❑ Widowed (skip to question 17)
- ❑ Other (specify) ______________

16. For how long have you been in this relationship?
Years _________ Months________

17. Do you have children?
If you answer "yes", please specify how many children you have.
- ❑ No (skip to question 20)
- ❑ Yes. I have _____child(ren)

18. Do you have children under 18 years of age?
If you answer "yes", please specify how many children under 18 years old you have.
- ❑ No
- ❑ Yes. I have _______ children under 18 years of age.

19. Do your children live in your home?
If you answer "yes", please specify how many children live in your home.
- ❑ No
- ❑ Yes. _____child(ren) live in my home

20. Are you currently employed or self-employed (working for pay)?
- ❑ No (skip to question 24)
- ❑ Yes

21. Are you currently working full-time (e.g., according to your employment contract)?
- ❑ No. I work part-time: _______hours per week.
- ❑ Yes, I work full time: _______hours per week.

22. After any taxes and contributions, what is your average monthly income from employment?
Please include any additional or extra or lump sum payment, such as bonuses, 13 month, Christmas or Summer pays.
- ❑ ____________ (skip to question 24)
- ❑ I prefer not to answer this question.

23. Please indicate your monthly income level after taxes and contributions.[1]

---

[1] The income brackets provided in this question were applied in the UK. Income brackets for other countries were adjusted to reflect differences in average incomes of the population. These values were based on the Survey of Health, Ageing and Retirement in Europe (SHARE).

Please include any additional or extra or lump sum payment, such as bonuses, 13 month, Christmas or Summer pays.

- ❑ Less than £800
- ❑ Between £800 and 1500
- ❑ Between £1500 and £4000
- ❑ More than £4000
- ❑ Prefer not to answer this question

24. What is your religion, if any?[2]

- ❑ Protestant
- ❑ Roman Catholic
- ❑ Eastern Orthodox
- ❑ Islam
- ❑ Jewish
- ❑ Buddhist
- ❑ Hindu
- ❑ Other (specify)
- ❑ I am not religious (skip to question 26)
- ❑ Prefer not to say

25. What is the importance of religion in your life?

- ❑ Not important at all
- ❑ Not too important
- ❑ Fairly important
- ❑ Very important

26. Skip to question 29 if you answered "stayed the same" to question 10

"**What health condition(s) have you been diagnosed with?**

Please select all that apply.

- ❑ A heart attack including myocardial infarction or coronary thrombosis or any other heart problem including congestive heart failure
- ❑ High blood pressure or hypertension
- ❑ High blood cholesterol
- ❑ A stroke or cerebral vascular disease
- ❑ Diabetes or high blood sugar
- ❑ Chronic lung disease such as chronic bronchitis or emphysema
- ❑ Cancer (please specify the type of cancer if known).
- ❑ Stomach or duodenal ulcer, peptic ulcer
- ❑ Parkinson disease
- ❑ Cataracts
- ❑ Hip fracture
- ❑ Other fractures
- ❑ Alzheimer's disease, dementia, organic brain syndrome, senility or any other serious memory impairment, please specify:
- ❑ Multiple sclerosis

[2] Question not asked in Italy, Sweden and the Netherlands due to ethical reasons

- ❑ Rheumatoid Arthritis
- ❑ Osteoarthritis, or other rheumatism
- ❑ Chronic kidney disease
- ❑ Traumatic brain injury
- ❑ HIV/AIDS
- ❑ Other condition(s) _________
- ❑ I have not been diagnosed with any conditions (skip to question 29)

27. Which one of these health conditions do you think causes the most limitations to you?
____

28. How long have you been living with this health condition?
Years ______ Months_____

29. Please tell me if you have any difficulty with these activities because of a physical, mental, emotional or memory problem. (Katz, 1983)

Please select all that apply.

- ❑ Dressing, including putting on shoes and socks
- ❑ Walking across a room
- ❑ Bathing or showering
- ❑ Eating, such as cutting up their food
- ❑ Getting in or out of bed
- ❑ Using the toilet, including getting up or down
- ❑ None of these

30. Who would you consider to be the person who provides most of the informal care for you?:

- ❑ Spouse / Partner
- ❑ Child
- ❑ Parent
- ❑ Sibling
- ❑ Daughter- / Son-in-law
- ❑ Uncle / Aunt
- ❑ Nephew/ Niece
- ❑ Cousin
- ❑ Friend
- ❑ Neighbour
- ❑ Other, please specify ______________

Let's focus on this person who provides the most amount of care for you. From now on, we will refer to this person as "your loved one".

31. How long have you been receiving informal care from your loved one?

- ❑ Less than a month: ________ week(s).
- ❑ Less than a year: _______ month(s).
- ❑ More than a year: _______ year(s).

Please fill in the following questions in relation to the **last week OR on a typical week** if last week was a holiday, or if you were away, or ill etc)

A limit of 126 hours has been set for the number of hours of care that you receive each week. The purpose of this limit is not to undervalue or underestimate the care that you receive. This limit has been set to allow your caregiver time (6 hours per day) for basic needs such as resting/sleeping and doing non-care related tasks.

32. During the last week, on how many days were you provided with care from your loved one?

- ❑ 0 days
- ❑ 1 day
- ❑ 2 days
- ❑ 3 days
- ❑ 4 days
- ❑ 5 days
- ❑ 6 days
- ❑ 7 days

33. Besides the care or support you receive from your loved one, do you receive care from other informal caregivers?

- ❑ No,
- ❑ Yes, from _________________________________ [number] other informal caregivers

34. Would you say that the help you receive from your caregiver(s) meets your needs?

- ❑ All the time
- ❑ Usually
- ❑ Sometimes
- ❑ Hardly ever

35. Do you share a household with your loved one?

- ❑ No
- ❑ Yes (skip to question 39)

36. Where do you live?

- ❑ In my own home
- ❑ In someone else's home
- ❑ In an assisted living facility or other residential setting where some care is provided
- ❑ In a nursing care or long-term care facility

37. How do you reach the place where your loved one lives?

- ❑ They walk to my home
- ❑ They cycle to my home
- ❑ They drive to my home
- ❑ They use public transportation (bus, train)
- ❑ If Other, please specify ______________

38. How far (in minutes) is the place you live from the home of your loved one?

- ❑ less than 15 minutes
- ❑ 15 to 30 minutes

- ❑ 30 to 60 minutes
- ❑ 60 to 90 minutes
- ❑ 90 to 120 minutes
- ❑ More than 120 minutes

Now we want to ask you about the services that you may have received from paid care worker(s) at home over the past three months.
These services are called home care services and include assistance with household activities and personal care, nursing care, setting services and overnight care.

39. **Besides the care or support you received from your family or friends, have you been provided with home care by one or more paid workers over the last month?**
    - ❑ No (skip to question 53)
    - ❑ Yes

40. **How many paid care workers are involved with your home care?**
    - ❑ 1
    - ❑ 2 (skip to question 46)
    - ❑ 3 (skip to question 46)
    - ❑ 4 (skip to question 46)
    - ❑ More than 4 (skip to question 46)

41. **Was the paid worker a migrant care worker?**
    "Migrant worker" refers to a person who is employed in a country that they migrated to. This includes not only people who migrated for the purpose of employment, but also those who migrated for other reasons (eg., family or education) and then gained employment at a later date.
    - ❑ No (skip to question 44)
    - ❑ Yes
    - ❑ Do not know (skip to question 44)

42. **Please fill in the follow table based on the situation of the migrant care worker**

Employed on a live-in basis?	Gender	Age	Level of education	Marital status
No	Female	18-24	Primary	Single (never married)
Yes	Male	25-34	Secondary	Married
	Non-binary /third gender	35-44	Post-secondary vocational education (e.g., further education college, apprenticeships etc.)	In a civil union/domestic partnership
	Do not know	45-54	Post secondary academic education (e.g., university)	Divorced

		55-64	Not listed or other	Widowed
		65-74	Do not know	Do not know
		75+		
		Do not know		

43. **What is the nationality of the migrant care worker?**
    They are a national/citizen of:
    ____

Skip to question 52 if you answered "yes" to question 41

44. **Please fill in the follow table based on the situation of the paid care worker**

Employed on a live-in basis?	Gender	Age	Level of education	Marital status
No	Female	18-24	Primary	Single (never married)
Yes	Male	25-34	Secondary	Married
	Non-binary /third gender	35-44	Post-secondary vocational education (e.g., further education college, apprenticeships etc.)	In a civil union/domestic partnership
	Do not know	45-54	Post secondary academic education (e.g., university)	Divorced
		55-64	Not listed or other	Widowed
		65-74	Do not know	Do not know
		75+		

		Do not know		

45. Does the paid care worker have a migration background?
"Migration background" refers to a person who has:
(a) migrated into their present country of residence; and/or
(b) previously had a different nationality from their present country of residence; and / or
(c) at least one of their parents previously entered their present country of residence as a migrant.

- ❑ No
- ❑ Yes
- ❑ Do not know

Skip to question 51 if you answered "no" or "do not know" to question 41

46. Were any of these paid workers a migrant worker?
"Migrant worker" refers to a person who is employed in a country that they migrated to. This includes not only people who migrated for the purpose of employment, but also those who migrated for other reasons (eg., family or education) and then gained employment at a later date.

- ❑ No (skip to question 49)
- ❑ Yes
- ❑ Do not know (skip to question 49)

47. Please fill in a separate row in the table for each individual worker. "Migration background" refers to a person who has:
(a) migrated into their present country of residence; and/or
(b) previously had a different nationality from their present country of residence; and / or
(c) at least one of their parents previously entered their present country of residence as a migrant.

	**Employed on a live-in basis?**	**Migrant worker ?**	**Migration background?**	**Gender**	**Age**	**Level of education**	**Marital status**
Care worker n. 1	No<br>Yes	No<br>Yes<br>Do not know	No<br>Yes<br>Do not know	Female<br>Male<br>Non-binary /third gender	18-24<br>25-34<br>35-44<br>45-54<br>55-64	Primary<br>Secondary<br>Post-secondary vocational education (e.g., further education	Single (never married)<br>Married<br>In a civil union/domestic partnership

				Do not know	65-74<br><br>75+<br><br>Do not know	college, apprenticeships etc.)<br><br>Post secondary academic education (e.g., university)<br><br>Not listed or other<br><br>Do not know	Divorced<br><br>Widowed<br><br>Do not know
Care worker n. 2	No<br><br>Yes	No<br><br>Yes<br><br>Do not know	No<br><br>Yes<br><br>Do not know	Female<br><br>Male<br><br>Non-binary /third gender<br><br>Do not know	18-24<br><br>25-34<br><br>35-44<br><br>45-54<br><br>55-64<br><br>65-74<br><br>75+<br><br>Do not know	Primary<br>Secondary<br>Post-secondary vocational education (e.g., further education college, apprenticeships etc.)<br>Post secondary academic education (e.g., university)<br>Not listed or other<br>Do not know	Single (never married)<br><br>Married<br><br>In a civil union/domestic partnership<br><br>Divorced<br><br>Widowed<br><br>Do not know
Care worker n. 3	No<br><br>Yes	No<br><br>Yes<br><br>Do not know	No<br><br>Yes<br><br>Do not know	Female<br><br>Male<br><br>Non-binary /third gender<br><br>Do not know	18-24<br>25-34<br>35-44<br>45-54<br>55-64<br>65-74<br>75+<br>Do not know	Primary<br><br>Secondary<br><br>Post-secondary vocational education (e.g., further education college,	Single (never married)<br><br>Married<br><br>In a civil union/domestic partnership<br><br>Divorced

						apprenticeships etc.)<br><br>Post secondary academic education (e.g., university)<br><br>Not listed or other<br><br>Do not know	Widowed<br><br>Do not know
Care worker n. 4	No<br><br>Yes	No<br><br>Yes<br><br>Do not know	No<br><br>Yes<br><br>Do not know	Female<br><br>Male<br><br>Non-binary /third gender<br><br>Do not know	18-24<br><br>25-34<br><br>35-44<br><br>45-54<br><br>55-64<br><br>65-74<br><br>75+<br><br>Do not know	Primary<br><br>Secondary<br><br>Post-secondary vocational education (e.g., further education college, apprenticeships etc.)<br><br>Post secondary academic education (e.g., university)<br><br>Not listed or other<br><br>Do not know	Single (never married)<br><br>Married<br><br>In a civil union/domestic partnership<br><br>Divorced<br><br>Widowed<br><br>Do not know

48. What is the nationality of the migrant care worker(s)?

They are a national/citizen of:
Care worker n. 1 __________
Care worker n. 2 __________
Care worker n. 3 __________
Care worker n. 4 __________

Skip to question 50 if you answered "yes" to question 46 and at least one paid care worker was not a migrant. If all care workers were migrant workers, skip to question 52

49. Please fill in a separate row in the table for each individual worker. "Migration background" refers to a person who has:

(a) migrated into their present country of residence; and/or

(b) previously had a different nationality from their present country of residence; and / or

(c) at least one of their parents previously entered their present country of residence as a migrant.

	**Employed on a live-in basis?**	**Migration background ?**	**Gender**	**Age**	**Level of education**	**Marital status**
Care worker n. 1	No<br>Yes	No<br>Yes<br>Do not know	Female<br>Male<br>Non-binary /third gender<br>Do not know	18-24<br>25-34<br>35-44<br>45-54<br>55-64<br>65-74<br>75+<br>Do not know	Primary<br>Secondary<br>Post-secondary vocational education (e.g., further education college, apprenticeships etc.)<br>Post secondary academic education (e.g., university)<br>Not listed or other<br>Do not know	Single (never married)<br>Married<br>In a civil union/domestic partnership<br>Divorced<br>Widowed<br>Do not know
Care worker n. 2	No<br>Yes	No<br>Yes<br>Do not know	Female<br>Male	18-24<br>25-34	Primary<br>Secondary<br>Post-secondary vocational	Single (never married)

			Non-binary /third gender<br><br>Do not know	35-44<br><br>45-54<br><br>55-64<br><br>65-74<br><br>75+<br><br>Do not know	education (e.g., further education college, apprenticeships etc.)<br>Post secondary academic education (e.g., university)<br>Not listed or other<br>Do not know	Married<br><br>In a civil union/domestic partnership<br><br>Divorced<br><br>Widowed<br><br>Do not know
Care worker n. 3	No<br><br>Yes	No<br><br>Yes<br><br>Do not know	Female<br><br>Male<br><br>Non-binary /third gender<br><br>Do not know	18-24<br>25-34<br>35-44<br>45-54<br>55-64<br>65-74<br>75+<br>Do not know	Primary<br><br>Secondary<br><br>Post-secondary vocational education (e.g., further education college, apprenticeships etc.)<br><br>Post secondary academic education (e.g., university)<br><br>Not listed or other<br><br>Do not know	Single (never married)<br><br>Married<br><br>In a civil union/domestic partnership<br><br>Divorced<br><br>Widowed<br><br>Do not know

Care worker n. 4	No<br><br>Yes	No<br><br>Yes<br><br>Do not know	Female<br><br>Male<br><br>Non-binary /third gender<br><br>Do not know	18-24<br><br>25-34<br><br>35-44<br><br>45-54<br><br>55-64<br><br>65-74<br><br>75+<br><br>Do not know	Primary<br><br>Secondary<br><br>Post-secondary vocational education (e.g., further education college, apprenticeships etc.)<br><br>Post secondary academic education (e.g., university)<br><br>Not listed or other<br><br>Do not know	Single (never married)<br><br>Married<br><br>In a civil union/domestic partnership<br><br>Divorced<br><br>Widowed<br><br>Do not know

Skip to question 51 if you answered "no" or "do not know" to question 46

50. Overall, how would you rate the home care services you receive from national/local care workers?[3]

- ❑ Very good
- ❑ Good
- ❑ Neither good or poor
- ❑ Poor
- ❑ Very poor

Skip to question 52 if you answered "yes" to question 46

51. Overall, how would you rate the home care services you receive?[4]

- ❑ Very good
- ❑ Good
- ❑ Neither good or poor

[3] Question not asked to participants in Sweden as the question was added after ethics approval in Sweden

[4] Question not asked to participants in Sweden as the question was added after ethics approval in Sweden

- ❑ Poor
- ❑ Very poor

Skip to question 53 if you answered "no" or "do not know" to question 41 or question 46

52. Overall, how would you rate the home care services you receive from migrant care workers?[5]

- ❑ Very good
- ❑ Good
- ❑ Neither good or poor
- ❑ Poor
- ❑ Very poor

53. Inclusion of Other in the Self Scale (IOS) (Aron et al., 1992)

Below there are seven pairs of circles: the level of overlap between the circles indicates how close you feel to your loved one. The greater the overlap, the closer you feel to your loved one. Please indicate the picture that best describes your relationship with your loved one.

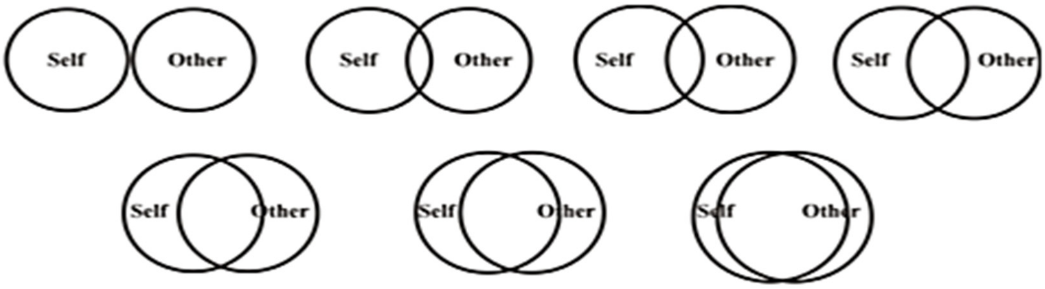

54. Partner-Specific Communal Motivation scale (CMS) (Lemay & Neal, 2013)

Keeping in mind your loved one, indicate the extent to which each statement applies to you.

	Not at all								Extremely
Helping me is a high priority for my loved one	1	2	3	4	5	6	7	8	9
My loved one could easily put my needs out of his/her thoughts	1	2	3	4	5	6	7	8	9
My loved one cares for me	1	2	3	4	5	6	7	8	9
My loved one cares about my well-being	1	2	3	4	5	6	7	8	9
My loved one could easily accept not helping me	1	2	3	4	5	6	7	8	9
My loved one would sacrifice very much to help me	1	2	3	4	5	6	7	8	9
My loved one would incur a large cost in order to help me	1	2	3	4	5	6	7	8	9

[5] Question not asked to participants in Sweden as the question was added after ethics approval in Sweden

My loved one cares for my needs	1	2	3	4	5	6	7	8	9
My loved one would go out of his/her way to help me	1	2	3	4	5	6	7	8	9
My loved one would be reluctant to sacrifice for me	1	2	3	4	5	6	7	8	9

55. Willingness to care scale (WTC) (Abell, 2001)

Receiving care from your loved one can sometimes be a difficult experience. Your loved ones may differ in the tasks they feel able to perform as well as you may differ in your willingness to accept a particular type of care task.

Please read each of the following questions and rate the extent to which you are willing or not to receive help from your loved one for each of these tasks.

How willing are you to accept:

instrumental help from your loved one? (e.g. having your meals prepared by your loved one, having your dishes washed, having your house cleaned, having your laundry done, etc.)	Completely unwilling	Somewhat unwilling	Not sure	Somewhat willing	Completely willing
emotional help from your loved one? (e.g. being listened by your loved one when you're sad, being encouraged by them when you feel hopeless, etc.)					
nursing help from your loved one? (e.g. helping you with dressing, feeding, washing, toileting, taking medicine, etc.)					

56. The World Health Organisation-Five Well-Being Index (WHO-5)

	Over the last two weeks	All of the time	Most of the time	More than half of the time	Less than half of the time	Some of the time	At no time
**1**	I have felt cheerful and in good spirits	5	4	3	2	1	0
**2**	I have felt calm and relaxed	5	4	3	2	1	0

**3**	I have felt active and vigorous	5	4	3	2	1	0
**4**	I woke up feeling fresh and rested	5	4	3	2	1	0
**5**	My daily life has been filled with things that interest me	5	4	3	2	1	0

57. EQ-5D-5L

Please select the ONE box that best describes your health TODAY.

MOBILITY

- ❑ I have no problems in walking about
- ❑ I have slight problems in walking about
- ❑ I have moderate problems in walking about
- ❑ I have severe problems in walking about
- ❑ I am unable to walk about

Please select the ONE box that best describes your health TODAY.

SELF-CARE

- ❑ I have no problems washing or dressing myself
- ❑ I have slight problems washing or dressing myself
- ❑ I have moderate problems washing or dressing myself
- ❑ I have severe problems washing or dressing myself
- ❑ I am unable to wash or dress myself

Please select the ONE box that best describes your health TODAY.

USUAL ACTIVITIES (e.g. work, study, housework, family or leisure activities)

- ❑ I have no problems doing my usual activities
- ❑ I have slight problems doing my usual activities
- ❑ I have moderate problems doing my usual activities
- ❑ I have severe problems doing my usual activities
- ❑ I am unable to do my usual activities

Please select the ONE box that best describes your health TODAY.

PAIN / DISCOMFORT

- ❑ I have no pain or discomfort
- ❑ I have slight pain or discomfort
- ❑ I have moderate pain or discomfort
- ❑ I have severe pain or discomfort
- ❑ I have extreme pain or discomfort

Please select the ONE box that best describes your health TODAY.

ANXIETY / DEPRESSION

- ❑ I am not anxious or depressed
- ❑ I am slightly anxious or depressed
- ❑ I am moderately anxious or depressed
- ❑ I am severely anxious or depressed

❑ I am extremely anxious or depressed

58. VAS

We would like to know how good or bad your health is TODAY.

This scale is numbered from 0 to 100.

100 means the best health you can imagine. 0 means the worst health you can imagine.

Please indicate on the scale how your health is TODAY.

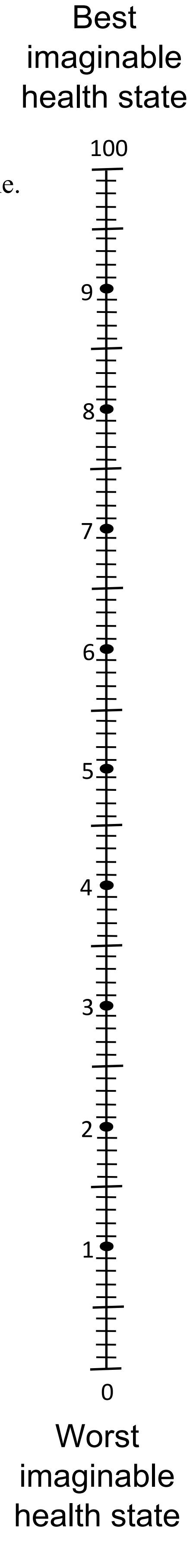

59. **10-item Center for Epidemiologic Studies Depression Scale (CESD-10)** (Andersen et al., 1994)

Below is a list of the ways you might have felt or behaved. Please indicate how often you have felt this way during the past week.

	**Rarely or none of the time (less than 1 day)**	**Some or a little of the time (1-2 days)**	**Occasionally or a moderate amount of time (3-4 days)**	**All of the time (5-7 days)**
I was bothered by things that usually don't bother me				
I had trouble keeping my mind on what I was doing				
I felt depressed				
I felt that everything I did was an effort				
I felt hopeful about the future				
I felt fearful				
My sleep was restless				
I was happy				
I felt lonely				
I could not "get going"				

60. **Dyadic Relationship Scale (DRS)** (Sebern et al. 2007)

This series of questions address some of the difficulties that people face as they receive care from a relative. We would like to know how receiving care from your loved one has affected your relationship with them during the past week. How much do you agree or disagree with the following statements?

**Because of helping my loved one:**	**Strongly agree**	**Agree**	**Neutral**	**Disagree**	**Strongly disagree**
I felt closer to my loved one than I have in a while.					
I have learned some good things about my loved one.					
I felt angry toward my loved one.					

I felt depressed because of my relationship with my loved one.					
I felt resentful toward my loved one.					
I have had more patience than I have had in the past.					
I have learned some good things about myself.					
I felt that my relationship with my loved one was strained.					
I have learned some nice things about other people in my life.					
Communication between my loved one and me has improved.					
I felt that my loved one made requests over and above what he/she needed.					

61. **Relationship Assessment Scale -** 1 item (Hendrick, 1988)

	**Not satisfied 1**	**2**	**3**	**4**	**Very satisfied 5**
In general, how satisfied are you with your relationship with your loved one?					

Module 1

62. **Revised Familism Scale (RFS)** (Losada et al., 2020)

The following questions examine family relationships. Please rate on the scale how much you agree or disagree with each statement:

	**Strongly disagree**	**Disagree**	**Neither agree nor disagree**	**Agree**	**Strongly agree**
1 - One should make great sacrifices in order to guarantee a good education for his/her children.					

2 - I would help within my means if a relative told me that they are in financial difficulty.					
3 - One should have the hope of living long enough to see his/her grandchildren grow up.					
4 - A person should share his/her home with uncles, aunts or first cousins if they are in need.					
5 - When someone has problems s/he can count on the help of relatives. (Generally)					
6 - When one has problems, one can count on the help of relatives. (Oneself/In relation to self)					
7 - One can count on help from his/her relatives to solve most problems.					
8 - Much of what a son or daughter does should be done to please the parents.					
9 - The family should consult close relatives (uncles, aunts) concerning its important decisions.					
10 - One should be embarrassed about the bad things done by his/her brothers or sisters.					
11 - Children should live in their parents' house until they get married.					
12 - A person should help his or her elderly parents in times of need, for example, help financially or share a house.					

13 - A person should live near his or her parents and spend time with them on a regular basis.					
14 - A person should always support members of the extender family, for example, aunts, uncles, and in-laws, if they are in need even if it is a big sacrifice.					
15 - A person should respect his or her older brothers and sisters regardless of their differences in views.					
16 - A person should be a good person for the sake of his or her family.					
17 - Children should obey their parents without question even if they believe they are wrong.					
18 - Parents and grandparents should be treated with great respect regardless of their differences in views					
19 - A person should often do activities with his or her immediate and extended families, for example, eat meals, play games, go somewhere together, or work on things together.					
20 - The family should control the behavior of children younger than 18.					
21 - A person should cherish time spent with his or her relatives.					

63. **The Brief Illness Perception Questionnaire (B-IPQ)** / Different format (Broadbent et al. 2006)

For the following questions, please select the number that best corresponds to your views:

How much does your illness affect your life?										
	1	2	3	4	5	6	7	8	9	10
no affect at all										severely affects my life
How long do you think your illness will continue?										
	1	2	3	4	5	6	7	8	9	10
a very short time										forever
How much control do you feel you have over your illness?										
	1	2	3	4	5	6	7	8	9	10
absolutely no control										extreme amount of control
How much do you think your treatment can help your illness?										
	1	2	3	4	5	6	7	8	9	10
not at all										extremely helpful
How much do you experience symptoms from your illness?										
	1	2	3	4	5	6	7	8	9	10
no symptoms at all										many severe symptoms
How concerned are you about your illness?										
	1	2	3	4	5	6	7	8	9	10
not at all concerned										extremely concerned
How well do you feel you understand your illness?										
	1	2	3	4	5	6	7	8	9	10
don't understand at all										understand very clearly

How much does your illness affect you emotionally? (e.g. does it make you angry, scared, upset or depressed)?<br>1 2 3 4 5 6 7 8 9 10<br>not at all affected emotionally ... extremely affected emotionally
Please list in rank-order the three most important factors that you believe caused your illness. The most important causes for me:<br>**1.** _<br>**2.** _<br>**3.** __

64. **Meaning in Life Questionnaire (MLQ)** (Steger et al., 2006)

Please take a moment to think about what makes your life and existence feel important and significant to you. Please respond to the following statements as truthfully and accurately as you can, and also please remember that these are very subjective questions and that there are no right or wrong answers. Please answer according to the scale below:

	**Absolutely untrue**	**Mostly Untrue**	**Somewhat Untrue**	**Can't Say True or False**	**Somewhat True**	**Mostly True**	**Absolutely True**
I understand my life's meaning.<br>I am looking for something that makes my life feel meaningful.							
I am always looking to find my life's purpose.							
My life has a clear sense of purpose.							
I have a good sense of what							

makes my life meaningful.							
I have discovered a satisfying life purpose.							
I am always searching for something that makes my life feel significant.							
I am seeking a purpose or mission for my life.							
My life has no clear purpose.							
I am searching for meaning in my life.							

65. **Portrait Values Questionnaire (PVQ-21)** (Schwartz et al., 2001)

Here we briefly describe some people. Please read each description and think about how much each person is or is not like you.

How much is this person like you?

	**very much like me**	**like me**	**somewhat like me**	**a little like me**	**not like me**	**not like me at all**
It is important to him/her to be rich. He/she wants to have a lot of money and expensive things.						

He/she thinks it is important that every person in the world should be treated equally. He/she believes everyone should have equal opportunities in life.						
It's very important to him/her to show his/her abilities. He/she wants people to admire what he/she does.						
It is important to him/her to listen to people who are different from him/her. Even when he/she disagrees with them, he/she still wants to understand them.						
It's very important to him/her to help the people around him/her. He/she wants to care for their well-being.						
Being very successful is important to him/her. He/she hopes people will recognize his/her achievements.						
It is important to him/her to get respect from others. He/She wants people to do what he/she says.						
It is important to him/her to be loyal to his/her friends. He/she wants to devote himself/herself to people close to him/her.						
He/she strongly believes that people should care for nature. Looking after the environment is important to him/her.						

Module 2

66. Over the past 3 months, how often did you feel that your loved one lacks information about your health condition?

For example, information about the course of your condition, medical care, medical exams results, etc.

- ❑ Never
- ❑ Rarely
- ❑ Sometimes
- ❑ Often
- ❑ Always

67. Over the past 3 months, how often did you feel that your loved one is not able to continue caring for you, due to your transportation needs?

Transportation needs refer to the assistance you might receive from your loved one to get to the services you use or activities (e.g. medical services, shopping or errands, recreational activities,

family or personal business, religious activities etc.), for example by driving you or helping you with using public transportation.

- ❑ Never
- ❑ Rarely
- ❑ Sometimes
- ❑ Often
- ❑ Always

68. Do you feel that your transportation needs, negatively impact your loved one's personal daily activities?

For example, housework, shopping, social outings, religious activities etc.

- ❑ Not at all
- ❑ A little
- ❑ Somewhat
- ❑ A lot

Skip to Question 74 if you answered "no" to question 35

Here are some questions about your living arrangements and the geographic distance between you and your loved one. Please read each of the questions carefully and choose the answer that best applies to you.

69. Over the past month, how often did your loved one visit you?

- ❑ Daily
- ❑ More than once a week
- ❑ Once a week
- ❑ Less than once a week

70. Do you think that the geographic distance between you and your loved one, is a barrier to their investment in your care?

- ❑ Not at all
- ❑ A little
- ❑ Somewhat
- ❑ A lot

71. Do you think that the travel time your loved one needs to get to you, negatively impacts their daily activities?

For example, housework, shopping, social outings, religious activities, etc.

- ❑ Not at all
- ❑ A little
- ❑ Somewhat
- ❑ A lot

72. Over the past 3 months, how often did you feel that your loved one is not able to continue caring for you, because you don't live together?

- ❑ Never
- ❑ Rarely
- ❑ Sometimes
- ❑ Often
- ❑ Always

73. Do you feel that the geographic distance between you and your loved one, negatively impacts your relationship with them?
    - ❑ Not at all
    - ❑ A little
    - ❑ Somewhat
    - ❑ A lot

74. The Relationship Structures Questionnaire of the Experiences in Close Relationships - Revised (ECR-RS)
(Fraley et al., 2011)

Please read each of the following statements carefully and rate the extent to which you believe each statement best describes your feelings about your loved one.
It helps to turn to my loved one in times of need.
strongly disagree 1 2 3 4 5 6 7 strongly agree
I usually discuss my problems and concerns with my loved one.
strongly disagree 1 2 3 4 5 6 7 strongly agree
I talk things over with my loved one.
strongly disagree 1 2 3 4 5 6 7 strongly agree
I find it easy to depend on my loved one.
strongly disagree 1 2 3 4 5 6 7 strongly agree
I don't feel comfortable opening up to my loved one.
strongly disagree 1 2 3 4 5 6 7 strongly agree
I prefer not to show my loved one how I feel deep down.
strongly disagree 1 2 3 4 5 6 7 strongly agree
I often worry that my loved one doesn't really care for me.
strongly disagree 1 2 3 4 5 6 7 strongly agree
I'm afraid that my loved one may abandon me.
strongly disagree 1 2 3 4 5 6 7 strongly agree
I worry that my loved one won't care about me as much as I care about them.
strongly disagree 1 2 3 4 5 6 7 strongly agree

---

75. The Pearlin Mastery Scale (Pearlin & Schooler, 1978)
On a scale of 1 to 4 with 1 meaning "Strongly Disagree" and 4 meaning "Strongly Agree", how strongly do you agree or disagree with these statements about yourself?

	Strong Disagree	Disagree	Agree	Strongly Agree
There is really no way I can solve some of the problems I have.				
Sometimes I feel that I'm being pushed around in life.				
I have little control over the things that happen to me.				

I can do just about anything I really set my mind to.				
I often feel helpless in dealing with the problems of life.				
What happens to me in the future mostly depends on me.				
There is little I can do to change many of the important things in my life.				

---

76. **Big-Five Inventory Extra Short Form (BFI-2-XS)** (Soto & John, 2017)

Here are a number of characteristics that may or may not apply to you. For example, do you agree that you are someone who likes to spend time with others? Please read each of the statements carefully and rate the extent to which you agree or disagree.
I am someone who...

	**Disagree strongly**	**Disagree a little**	**Neutral; no opinion**	**Agree a little**	**Agree strongly**
Tends to be quiet.					
Is compassionate, has a soft heart.					
Tends to be disorganized.					
Worries a lot.					
Is fascinated by art, music, or literature.					
Is dominant, acts as a leader.					
Is sometimes rude to others.					
Has difficulty getting started on tasks.					

Tends to feel depressed, blue.					
Has little interest in abstract ideas.					
Is full of energy.					
Assumes the best about people.					
Is reliable, can always be counted on.					
Is emotionally stable, not easily upset.					
Is original, comes up with new ideas.					

---

77. **Toronto Empathy Questionnaire (TEQ)** (Spreng et al., 2009)

Below is a list of statements. Please read each of the statements carefully and rate how frequently you feel or act in the manner described. There are no right or wrong answers or trick questions. Please answer each question as honestly as you can.

	**Never**	**Rarely**	**Sometimes**	**Often**	**Always**
When someone else is feeling excited, I tend to get excited too.					
Other people's misfortunes do not disturb me a great deal.					
It upsets me to see someone being treated disrespectfully.					
I remain unaffected when someone close to me is happy.					

I enjoy making other people feel better.					
I have tender, concerned feelings for people less fortunate than me.					
When a friend starts to talk about his\her problems, I try to steer the conversation towards something else.					
I can tell when others are sad even when they do not say anything.					
I find that I am “in tune” with other people’s moods.					
I do not feel sympathy for people who cause their own serious illnesses.					
I become irritated when someone cries.					
I am not really interested in how other people feel.					
I get a strong urge to help when I see someone who is upset.					

When I see someone being treated unfairly, I do not feel very much pity for them.					
I find it silly for people to cry out of happiness.					
When I see someone being taken advantage of, I feel kind of protective towards him/her.					

Module 3

78. **Perception of Collaboration Questionnaire (PCQ)** (Berg et al., 2008)

Here are some questions about perceptions of collaboration between you and your loved one. Please indicate the extent to which you agree or disagree with that statement

	Strongly disagree	Disagree	Neutral	Agree	Strongly agree
I view working together with my loved one as necessary as it is harder for me to solve problems and make decisions by myself					
I make better decisions when my loved one and I work together					
My loved one and I always work together to deal with					

care-related decisions					
Nearly every day my loved one and I work together to make care-related decisions					
It is rare for my loved one and I to share care-related tasks and make decisions together					
I enjoy the support and encouragement I receive when I work together with my loved one					
Working together with my loved one is useful as he/she makes up for things that I don't do well					
Solving everyday problems and making decisions together with my loved one brings us closer together					
I dislike getting my loved one's assistance as it makes me feel incompetent					

79. Dyadic Coping Inventory (DCI) - stress communication subscale (Bodenmann, 2000)
Please respond to any item by marking the appropriate case, which is fitting to your personal situation.
This section is about how you communicate your stress with your loved one:

	Very rarely	Rarely	Sometimes	Often	Very often
I let my loved one know that I appreciate his/her practical support, advice, or help					
I ask my loved one to do things for me when I have too much to do					
I show my loved one through my behaviour when I am not doing well or when I have problems					
I tell my loved one openly how I feel and that I would appreciate his/her support					

This section is about how your loved one communicates when he/she is feeling stressed:

	Very rarely	Rarely	Sometimes	Often	Very often
My loved one lets me know that he/she appreciates my practical support, advice, or help					
My loved one asks me to do things for					

him/her when he/she has too much to do					
My loved one shows me through his/her behaviour that he/she is not doing well or when he/she has problems					
My loved one tells me openly how he/she feels and that he/she would appreciate my support					

80. Mutuality scale (MS) (Archbold et al., 1990)

Now we would like you to let us know how you and your loved one feel about each other at the current time.

	Not at all	A little	Some	Quite a bit	A great deal
To what extent do the two of you see eye to eye (agree on things)?					
How close do you feel to your loved one?					
How much do you enjoy sharing past experiences with your loved one?					
How much does your loved one express					

feelings of appreciation for you and the things you do?					
How attached are you to your loved one?					
How much does your loved one help you?					
How much do you like to sit and talk with your loved one?					
How much love do you feel for your loved one?					
To what extent do the two of you share the same values?					
When you really need it, how much does your loved one comfort you?					
How much do the two of you laugh together?					
How much do you confide in your loved one?					
How much emotional support does					

your loved one give you?					
To what extent do you enjoy the time the two of you spend together?					
How often does your loved one express feelings of warmth toward you?					

81. The perceived partner responsiveness scale (PPRS) (Reis et al., 2018)

Please answer the following questions about your loved one.

My loved one usually:

	Not at all true		Somewhat true		Moderately true		Very true		Completely true
really listens to me									
is responsive to my needs									
sees the "real" me									
"gets the facts right" about me									
understands me									
is on the “same wavelength” with me									
knows me well									
esteems me, shortcomings and all									

values and respects the whole package that is the "real" me									
expresses liking and encouragement for me									
seems interested in what I am thinking and feeling									
values my abilities and opinions									

82. Social Support List (SSL) (Kempen & Van Eijk, 1995; Van Sonderen, 1993)
The next questions concern the help and support you provide to your loved one. Please keep your loved one in mind.
How often do you:

	Seldom or never	Now and then	Quite often	Very often
Provide attention to your loved one				
Provide support to your loved one				
Show that you appreciate your loved one				
Provide instrumental support to your loved one				
Keep your loved one company				
Provide information and advice to your loved one				

React in a distant manner to your loved one				
Do not keep your appointments with your loved one				
Make disapproving remarks to your loved one				
Blame or holding things against your loved one				
Treat your loved one unfairly				
Make unreasonable demands to your loved one				
Interfere too much with your loved one				

The next questions concern the help and support you receive from your loved one. Please keep your loved one in mind.
How often does your loved one:

	Seldom or never	Now and then	Quite often	Very often
Provide attention to you				
Provide support to you				
Show that he/she appreciates you				
Provide instrumental support to you				
Keep you company				

Provide information and advice to you				
React in a distant manner to you				
Do not keep his/her appointments with you				
Make disapproving remarks to you				
Blame or holding things against you				
Treat you unfairly				
Make unreasonable demands to you				
Interfere too much with you your loved one				

Module 4

83. Do you provide financial compensation to your loved one for the care that you are provided with?

- ❑ No
- ❑ Yes: ____ net per month.

84. Do you provide financial compensation to another family member for the care that you are provided with?

- ❑ No
- ❑ Yes: ____ net per month.

85. Do you receive any of the following benefits?

Benefit	Do you receive this? Yes/No	Approx. amount per month

Cash benefits (i.e. care allowance, personal budget, attendance allowance, cash domiciliary allowance, personal assistance benefits, sickness benefits, disability/invalidity/incapacity benefits, or any other cash benefits you might have received to help you pay for the care you need).		
Tax benefits such as exemptions, deductions, and credits (i.e council tax reduction, deductions from income tax, etc.)		
Voucher programs (service vouchers)		

Skip to question 90 if you answered no to question 39

Skip to question 89 if you selected "no" or "do not know" to question 41 or 46

86. Were you involved in the decision to hire a migrant worker?

- ❑ No
- ❑ Yes

87. Why was it decided to hire a migrant worker?

Please select all that apply.

- ❑ Economically convenient
- ❑ It is useful for both myself and my loved one
- ❑ Migrant workers provide care tasks that local staff are not able to provide (for example late night shifts/weekends).
- ❑ It is common practice
- ❑ To avoid living in a residential facility
- ❑ Do not know
- ❑ If Other, please specify ______________

88. How did you access services by a migrant care worker?[6]

Please select all that apply

- ❑ Through recommendations from family or friends
- ❑ Through a care or recruitment agency
- ❑ Through a church or religious organization
- ❑ Through a government programme
- ❑ Through a non-profit organization
- ❑ Through social media
- ❑ Other, please specify:

Skip to question 90 if you selected "yes" to question 41 or 46

[6] Question not asked to participants in Sweden as the question was added after ethics approval in Sweden

89. Why was it decided not to hire a migrant worker?

Please select all that apply.

- ❑ I would not trust a migrant worker to take care of me
- ❑ Not able to afford this service
- ❑ I prefer that the care worker is a national citizen
- ❑ My loved one refused this solution
- ❑ Do not know
- ❑ If Other, please specify

90. The next questions will ask about the services that you may have used in the 6 months.

Which of the following services have you received or accessed in the past 6 months?

Select all that apply.

Name of service	Have you received (the service) in the past 6 months?	How many times have you used this service in the past 6 months?
Emotional, psychological or social support service		
Occupational therapy (in the home or at a clinic).		
Physiotherapy (in the home or at a clinic)		
Medical and nursing services (in the home or at a clinic)		
Transport services		
Organizing and managing care and support		
Telecare (i.e alarm buttons, help lines, bed monitors, video communication systems, fall and movement detectors, smartphone apps or any other technologies that assist		

your loved one to continue living at home).		

91. *Skip to question 92 if you did not select any services in question 90.*

Of those services you have used which would you rate as the most important to you?

- ❑ Emotional, psychological or social support service
- ❑ Occupational therapy
- ❑ Physiotherapy
- ❑ Medical and nursing
- ❑ Services Transport services
- ❑ Organizing and managing care and support
- ❑ Telecare

92. Have you used any assistive devices/aids (devices and aids that help you live more independently such as communication systems, mobility aids, help lines, smartphone apps, etc.)?

- ❑ No
- ❑ Yes

93. Have you been in a daycare facility or a residential or nursing home in the past 6 months?

- ❑ No
- ❑ Yes

The next questions will ask about any financial costs that you may have had as a result of your need for help. These will be called “out-of-pocket” expenses. Please do not count expenses that are covered or reimbursed by a health or social insurance.

Skip to question 96 if you answered “no” to question 39

94. In the last six months, did you pay anything yourself for the paid or professional help you receive in your home without getting reimbursed by your health or long-term care insurance?

- ❑ No
- ❑ Yes

95. How much do you pay in a typical month for personal care, domestic tasks, meals on wheels or other help you receive in your own home?

____

Skip to question 98 if you answered "no" to question 93 or did not select "In a nursing care or long-term care facility" for question 36

96. In the last six months, did you pay anything yourself for nursing home and/or daycare facility stays without getting reimbursed by your health or long-term care insurance?

- ❑ No (skip to question 98)
- ❑ Yes

97. Can you tell me about how much you pay in a typical month for your nursing home and/or daycare facility stays?

____

98. Skip to question 100 if you answered "no" to question 92

In the last six months, did you pay anything yourself for assistive devices and aids without getting reimbursed by your health or long-term care insurance?

- ❑ No (skip to end of questionnaire)
- ❑ Yes

99. Can you tell me about how much you pay in a typical month for assistive devices and aids?

____

100. Have you had any assistance in filling out the survey?

- ❑ No
- ❑ Yes, my loved one helped me fill in the survey
- ❑ Yes, someone other than my loved one helped me fill in the survey

Thank you for completing our survey.

We very much appreciate the information that you have provided and your time taken in completing this survey.

You can find the latest updates on our project at: https://www.entwine-icohort.eu

If you have any further questions on the survey, please send us an email at: entwinestudy@bangor.ac.uk
