# Supplementary material for: A Multinational Longitudinal Study Incorporating Intensive Methods to Examine Caregiver Experiences in the Context of Chronic Health Conditions: Protocol of the ENTWINE-iCohort
Source: Int J Environ Res Public Health. 2022 Jan 12;19(2):821. doi: 10.3390/ijerph19020821 (PMC8775526; doi:10.3390/ijerph19020821)
Supplement: Supplementary file 1 [file ijerph-19-00821-s001.zip › Supplementary File S8. ENTWINE-iCOHORT Diary Study Weekly Measures-Caregiver version.pdf]

ENTWINE-iCOHORT DIARY STUDY WEEKLY MEASURES

Caregiver version

Good afternoon! Welcome to the ENTWINE weekly questionnaire for week N°:

1. How do you feel?

Right now I feel:

- Determined
- Attentive
- Alert
- Inspired
- Active
- Afraid
- Nervous
- Upset
- Ashamed
- Hostile
- Blue/sad
- Appreciated
- Grateful
- Resentful

0=not at all

4=extremely

2. Do you feel that caring for your loved one is a burden right now?

0=not at all

4=extremely

3. How satisfied are you with caregiving right now?

0=not at all

4=extremely

4. How satisfied are you with your relationship with you loved one right now?

0=not at all

4=extremely

5. How close do you feel to your loved one right now?

0=not at all

4=extremely

6. With whom do you feel most connected right now?

   Please select all that apply

   - partner
   - a family member (children, parents, siblings...)
   - a friend
   - a colleague
   - a care professional
   - other

7. How willing are you to look after your loved one's needs right now?

0=not at all

10=extremely

8. How interested are you in caregiving?

0=not at all

10=extremely

9. Would you prefer to do something else?

0=not at all

10=extremely

10. Have you provided any of these care tasks to your loved one today?

    Please select all that apply

    - Household activities (e.g., preparing meals, washing the dishes, cleaning the house, doing laundry)

    - Personal care (e.g., dressing, feeding, washing and toileting)

    - Practical support (e.g., taking your loved one to a medical appointment, organising financial and administrative matters)

- Emotional support (e.g., listening to your loved one when they're sad, encouraging them when they feel hopeless)

- Companionship (e.g., talking, reading, watching TV, just 'being there')

- None of them

- Other, please specify:

11. What kind of tasks have you performed over the past week?
    Please select all that apply and the number of hours you have spent on each.

- Household activities
  Number of hours

- Personal Care
  Number of hours

- Practical support
  Number of hours

- Emotional support
- Number of hours

- Companionship
  Number of hours

- None of them

- Other, please specify:

12. How willing have you been to look after your loved one's needs in the past week?

0=not at all
10=extremely

13. This past week my loved one's health condition has interfered with my:
- Physical activities (e.g., cleaning, lifting objectives, sports)
- Social activities (e.g., visit friends and family)

- Mental activities (e.g., reading, working)
- Quality of time my loved one and I spent together (e.g., talking, listening to music, watching a movie…)

0=not at all
4=extremely

14. How has your loved one's health status been in the past week?

0=poor
4=excellent

15. How has your health been in the past week?

0=poor
4=excellent

16. How much meaning have you found in caregiving in the past week?

0 = absolutely meaningless
4 = absolutely meaningful

17. How have you spent time with your loved one in the past week?

Please select all that apply

- Activities together
- Contact via telephone
- Contact via sms, chat, email, social media
- Together but involved in different activities
- Other, please specify:

18. How tense have your interactions with your loved one been in the past week?

0=not at all
4= extremely

19. How enjoyable have your interactions with your loved one been in the past week?

0=not at all
4=extremely

20. This past week:

- I have ignored issues arising from caregiving
- I have avoided my loved one
- I have withdrawn from the caregiving situation
- I have helped my loved one

- I have listened to my loved one
- I have given him/her advice
- My loved one and I have worked together as a team
- My loved one and I have discussed the best way to deal with caregiving
- My loved one and I have solved caregiving issues together
- I have taken charge of the caregiving
- I have told my loved one what to do
- I have decided what to do (without consulting my loved one)
- I have avoided disagreeing with my loved one
- I have acted more positive than I felt
- I have withheld potentially upsetting information with my loved one

0=not at all
4=extremely

21. How well do you think you have dealt with issues arising from caregiving in the past week?

0=not at all
4=extremely

22. How much have you revealed your thoughts and feelings about caregiving to your loved one in the past week?

23. How much has your loved one revealed thoughts and feelings about caregiving to you in the past week?

0=not at all
4=extremely

24. This past week my loved one:

- Has been understanding
- Has been loving
- Has been supportive
- Has taken me seriously
- Has been critical
- Has talked about something else
- Has showed appreciation to my caregiving role
- Has been an overly demanding person to care for
- Has tried to manipulate me

- Has caused my family and me much aggravation
- Has made unnecessary requests of me for care

0=not at all
4=extremely

25. How satisfied are you with the way your loved one has responded to you in the past week?

0=not at all
4=extremely

26. How balanced has your relationship with your loved one been in terms of give-and-take in the past week? (e.g., doing things for each other, talking about each other's problems, listening to each other, receiving/providing help and attention)

- My loved one has done a lot more for me than I have done for him/her
- My loved one has done more for me than I have done for him/her
- My loved one has done as much for me as I have done for him/her
- My loved one has done a lot less for me than I have done for him/her
- My loved one has done a lot less for me than I have done for him/her

Thank you for completing our survey!

We very much appreciate the information that you have provided and your time taken in completing this survey.

We will send you the following questionnaire next week!

Remember, we ask the same questions every week on purpose! We need to find out how your experiences change over time.
