# Supplementary material for: A Multinational Longitudinal Study Incorporating Intensive Methods to Examine Caregiver Experiences in the Context of Chronic Health Conditions: Protocol of the ENTWINE-iCohort
Source: Int J Environ Res Public Health. 2022 Jan 12;19(2):821. doi: 10.3390/ijerph19020821 (PMC8775526; doi:10.3390/ijerph19020821)
Supplement: Supplementary file 1 [file ijerph-19-00821-s001.zip › Supplementary File S9. ENTWINE-iCOHORT Diary Study Weekly Measures-Care Recipient.pdf]

ENTWINE-iCOHORT DIARY STUDY WEEKLY MEASURES

Care recipient version

Good afternoon! Welcome to the ENTWINE weekly questionnaire for week N°:

1. How do you feel?

Right now I feel:
- Determined
- Attentive
- Alert
- Inspired
- Active
- Afraid
- Nervous
- Upset
- Ashamed
- Hostile
- Blue/sad
- Appreciated
- Grateful
- Resentful

0=not at all
4=extremely

2. How close do you feel to your loved one right now?

0=not at all
4=extremely

3. With whom do you feel most connected right now?

Please select all that apply
- partner
- a family member (children, parents, siblings...)
- a friend
- a colleague
- a care professional
- other

4. How satisfied are you with your relationship with you loved one right now?

0=not at all

4=extremely

5. How willing are you to look after your loved one's needs right now?

0=not at all

10=extremely

6. How much do you think your loved one is willing to look after your needs right now?

0=not at all

10=extremely

7. How interested is your loved one in providing care?

0=not at all

10=extremely

8. Do you think your loved one would prefer to do something else?

0=not at all

10=extremely

9. Have you received any of these care tasks to your loved one today?
   Please select all that apply

- Household activities (e.g., your loved one has prepared meals, washed the dishes, cleaned the house, done the laundry)
- Personal care (e.g., your loved one has helped you in dressing, feeding, washing and toileting)
- Practical support (e.g., your loved one has led you to a medical appointment, organised financial and administrative matters)
- Emotional support (e.g., your loved one has listened to you when you were sad, encouraged you when you feel hopeless)
- Companionship (e.g., your loved one has been talking, reading, watching TV, just 'being there')
- None of them
- Other, please specify:

10. What kind of tasks has your loved one provided to you over the past week?
    Please select all that apply and the number of hours your loved one has spent on each.

- Household activities
  Number of hours

- Personal Care
  Number of hours

- Practical support
  Number of hours

- Emotional support
- Number of hours

- Companionship
  Number of hours

- None of them

- Other, please specify:

11. How willing have you been to look after your loved one's needs in the past week?

0=not at all

10=extremely

12. How much do you think your loved one has been willing to look after your needs in the past week?

13. This past week my health condition has interfered with my:
- Physical activities (e.g., cleaning, lifting objectives, sports)
- Social activities (e.g., visit friends and family)
- Mental activities (e.g., reading, working)
- Quality of time my loved one and I spent together (e.g., talking, listening to music, watching a movie…)

0=not at all

4=extremely

14. How has your loved one's health status been in the past week?

0=poor

4=excellent

15. How has your health been in the past week?

0=poor

4=excellent

16. How have you spent time with your loved one in the past week?

Please select all that apply

- Activities together
- Contact via telephone
- Contact via sms, chat, email, social media
- Together but involved in different activities
- Other, please specify:

17. How tense have your interactions with your loved one been in the past week?

0=not at all

4= extremely

18. How enjoyable have your interactions with your loved one been in the past week?

0=not at all

4=extremely

19. This past week:

- My loved one has ignored issues arising from caregiving
- My loved one has avoided me
- My loved one has withdrawn from the caregiving situation
- My loved one has helped me
- My loved one has listened to me
- My loved one has given me advice
- My loved one and I have worked together as a team
- My loved one and I have discussed the best way to deal with caregiving
- My loved one and I have solved caregiving issues together
- My loved one has taken charge of the caregiving
- My loved one has told me what to do
- My loved one has decided what to do without consulting me
- I have avoided disagreeing with my loved one
- I have acted more positive than I felt
- I have withheld potentially upsetting information with my loved one

0=not at all
4=extremely

20. How well do you think you have dealt with issues arising from carein the past week?

0=not at all
4=extremely

21. How much have you revealed your thoughts and feelings about caregiving to your loved one in the past week?

22. How much has your loved one revealed thoughts and feelings about caregiving to you in the past week?

0=not at all
4=extremely

23. This past week my loved one:

- Has been understanding
- Has been loving
- Has been supportive
- Has taken me seriously
- Has been critical
- Has talked about something else

0=not at all
4=extremely

24. How satisfied are you with the way your loved one has responded to you in the past week?

0=not at all
4=extremely

25. How balanced has your relationship with your loved one been in terms of give-and-take in the past week? (e.g., doing things for each other, talking about each other's problems, listening to each other, receiving/providing help and attention)

- My loved one has done a lot more for me than I have done for him/her
- My loved one has done more for me than I have done for him/her
- My loved one has done as much for me as I have done for him/her
- My loved one has done a lot less for me than I have done for him/her
- My loved one has done a lot less for me than I have done for him/her

Thank you for completing our survey!

We very much appreciate the information that you have provided and your time taken in completing this survey.

We will send you the following questionnaire next week!

Remember, we ask the same questions every week on purpose! We need to find out how your experiences change over time.
