# Supplementary figures and images for: A Multinational Longitudinal Study Incorporating Intensive Methods to Examine Caregiver Experiences in the Context of Chronic Health Conditions: Protocol of the ENTWINE-iCohort
Source: Int J Environ Res Public Health. 2022 Jan 12;19(2):821. doi: 10.3390/ijerph19020821 (PMC8775526; doi:10.3390/ijerph19020821)

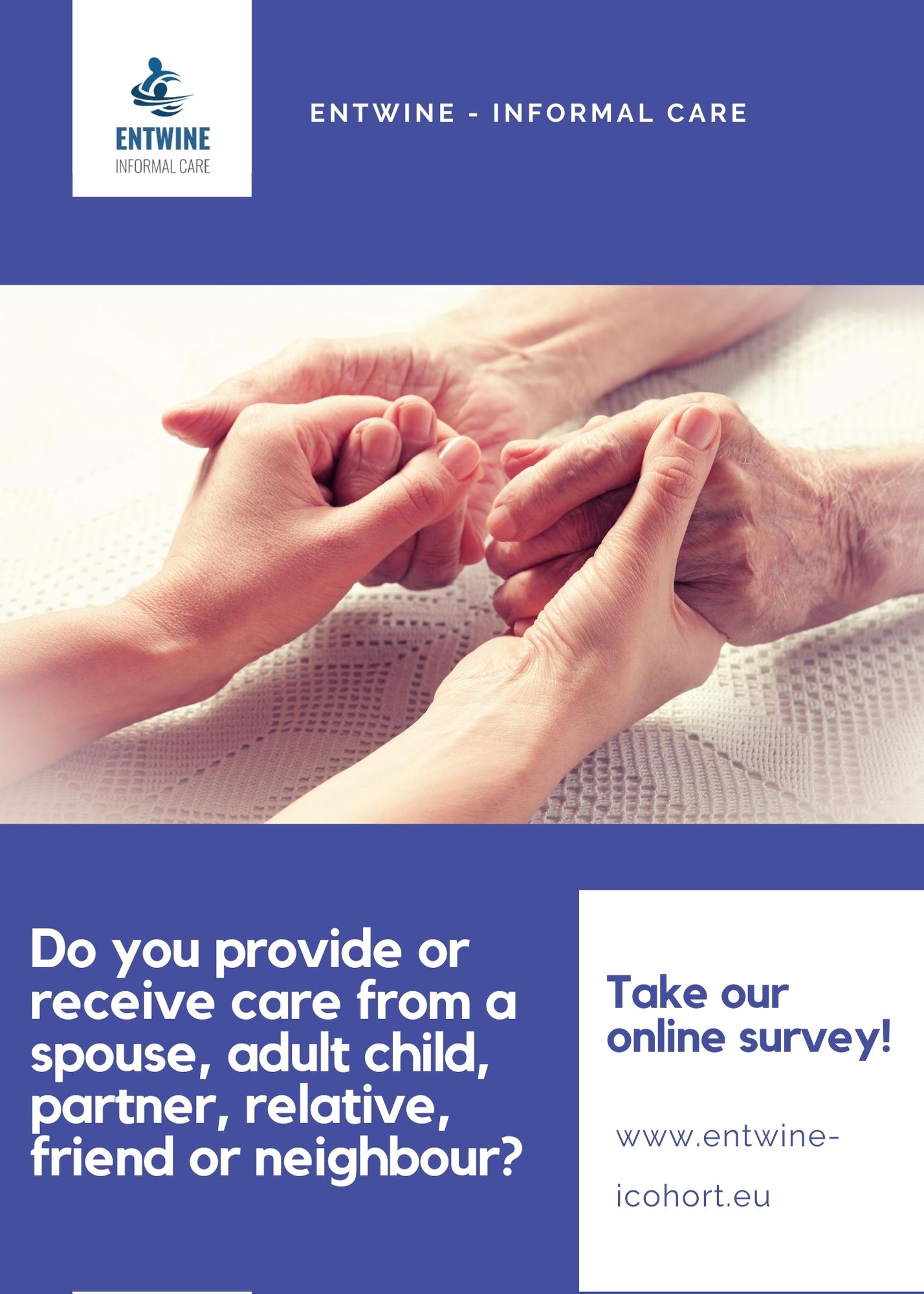

Supplement: Supplementary file 1 [file ijerph-19-00821-s001.zip › Supplementary File S1. ENTWINE-iCohort exemplary fliers (1¿C3).jpg]
